# piRNAs regulate a Hedgehog germline-to-soma pro-aging signal

**DOI:** 10.1038/s43587-022-00329-2

**Published:** 2023-01-09

**Authors:** Cheng Shi, Coleen T. Murphy

**Affiliations:** 1 Department of Molecular Biology and Lewis-Sigler Institute for Integrative Genomics, Princeton University, Princeton, NJ USA; 2 Present Address: Department of Biological Sciences, University of New Orleans, New Orleans, LA USA

**Keywords:** Gene expression, Gene regulation, Ageing

## Abstract

The reproductive system regulates somatic aging through competing anti- and pro-aging signals. Germline removal extends somatic lifespan through conserved pathways including insulin and mammalian target-of-rapamycin signaling, while germline hyperactivity shortens lifespan through unknown mechanisms. Here we show that mating-induced germline hyperactivity downregulates piRNAs, in turn desilencing their targets, including the Hedgehog-like ligand-encoding genes *wrt-1* and *wrt-10*, ultimately causing somatic collapse and death. Germline-produced Hedgehog signals require PTR-6 and PTR-16 receptors for mating-induced shrinking and death. Our results reveal an unconventional role of the piRNA pathway in transcriptional regulation of Hedgehog signaling and a new role of Hedgehog signaling in the regulation of longevity and somatic maintenance: Hedgehog signaling is controlled by the tunable piRNA pathway to encode the previously unknown germline-to-soma pro-aging signal. Mating-induced piRNA downregulation in the germline and subsequent Hedgehog signaling to the soma enable the animal to tune somatic resource allocation to germline needs, optimizing reproductive timing and survival.

## Abstract (web summary)

This study identifies a new germline-to-soma aging signal tuned by mating in *Caenorhabditis* *elegans*. The authors find that germline piRNAs influence longevity and somatic maintenance by transcriptionally regulating germline-to-soma Hedgehog signaling.

## Main

Longevity is plastic and is influenced by external factors, for example, diet, and internal factors such as reproductive demands. Communication between the germline and soma allows a coordinated response to physiological and environmental challenges. Animals couple nutrient availability to reproduction^1^, a typical example of soma-to-germline communication, through conserved signaling pathways, including the insulin, AMP-activated protein kinase (AMPK), and mammalian target-of-rapamycin (mTOR) pathways^2–6^. Conversely, the status of worm, fly, mouse, and human germlines influence their somatic tissues: germline removal and ovary transplantation extend lifespan^7–11^, while germline hyperactivity decreases lifespan and leads to dramatic changes in somatic physiology in animals across great evolutionary distances^12–14^. While these studies in animals ranging from invertebrates to humans support the existence of a pro-aging signal from the germlines, the nature and identity of that signal remains elusive.

Removal of the germline extends lifespan, increases fat accumulation, and enhances resistance to various stresses^7,10,15–18^. However, concomitant removal of the somatic gonad eliminates lifespan extension and other somatic benefits, suggesting the existence of two opposing signals: a germline pro-aging signal and a somatic gonad prolongevity signal^7,19^. The somatic gonad prolongevity signal pathway has been characterized using germlineless worms^20–28^. Dafachronic acids, insulin, mTOR, and steroid signaling are required in the soma for germline loss-mediated lifespan regulation, suggesting that they mediate the somatic gonad prolongevity effect^11,19–21,24,29,30^. By contrast, the identity of the initial pro-aging signal originating from the germline remains elusive. Identifying this pro-aging signal is critical for understanding how animals tune their aging rates in response to germline activity and reproductive needs.

While most studies of the influence of germline on lifespan have compared germlineless animals with intact, unmated animals^7,19^, less is known about the role of the hyperactive germline. Mating accelerates germline proliferation and ultimately leads to somatic collapse, shrinking and early death^12,13^, suggesting that the germline pro-aging signal is substantially amplified by mating. Therefore, mated worms are an ideal system in which to identify the mysterious germline pro-aging signal.

We set out to identify the underlying mechanism of hyperactive germline-induced shrinking and early death. Our transcriptional analyses of isolated germlines revealed that a specific subset of piRNAs is downregulated in response to mating, de-repressing Hedgehog-related genes. Hedgehog signaling communicates the status of the germline to somatic cells, resulting in mating-induced shrinking and early death. Thus, somatic responses to germline hyperactivity are tuned by piRNA regulation of an important developmental signaling pathway.

## Results

### Mating induces transcriptional changes in the germline

**Fig. 1:**
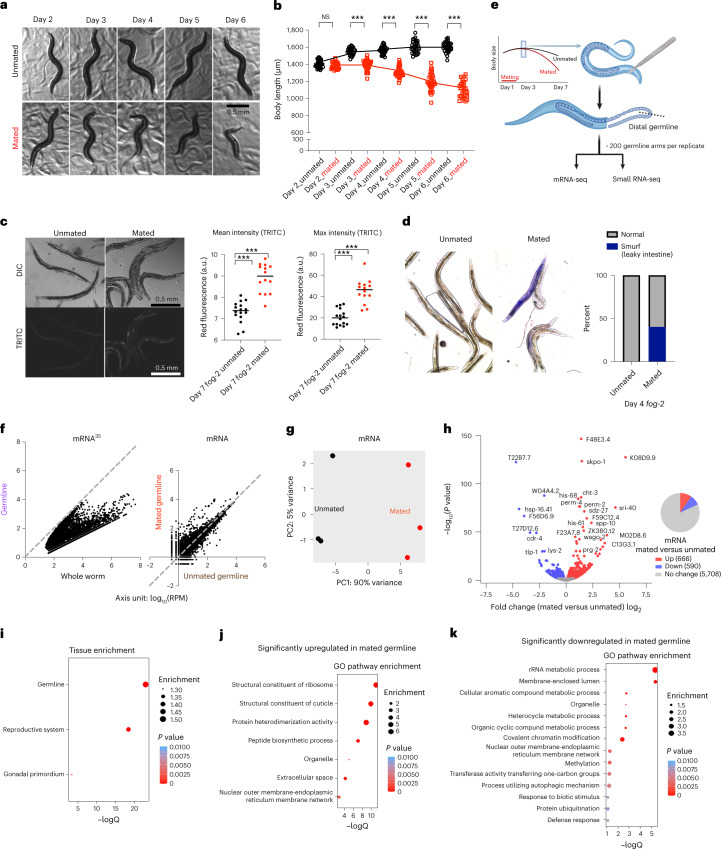
Mating induces shrinking and significant transcriptional changes in the germline. **a**, Representative images of the unmated and mated hermaphrodites from Day 2–6 of adulthood. Mating occurs on Day 1 for 24 h. **b**, Length of unmated and mated *fog-2* hermaphrodites. Day 2 unmated, 1,407 ± 45 μm (*n* = 30); Day 2 mated, 1,386 ± 39 μm (*n* = 30); *P* = 0.0642. Day 3 unmated, 1,552 ± 41 μm (*n* = 30); Day 3 mated, 1,392 ± 59 μm (*n* = 30); *P* < 0.0001. Day 4 unmated, 1,568 ± 44 μm (*n* = 30); Day 4 mated, 1,309 ± 59 μm (*n* = 30); *P* < 0.0001. Day 5 unmated, 1,604 ± 69 μm (*n* = 30); Day 5 mated, 1,194 ± 100 μm (*n* = 30); *P* < 0.0001. Day 6 unmated, 1,603 ± 59 μm (*n* = 30); Day 6 mated, 1,102 ± 95 μm (*n* = 29); *P* < 0.0001; ***P < 0.0001, NS, no statistical difference. Body size data are presented as mean body length ± s.d. for all body size measurements in this study; *n*, number of biologically independent animals for all body size measurements. Two-tailed *t*-test was used for all body size measurement comparisons in this study. **c**, Autofluorescence (red) is significantly increased by mating. Left, representative images of Day 7 mated and unmated *fog-2* hermaphrodites; right, quantification of mean and maximum red autofluorescence (via tetramethylrhodamine isothiocyanate (TRITC) filterset). Unmated mean, 7.3 ± 0.5 (*n* = 16); mated mean, 8.9 ± 0.7 (*n* = 14) arbitrary units (a.u.); *P* < 0.0001. Unmated max, 20.4 ± 7.6 (*n* = 16); mated max, 45.1 ± 11.5 (*n* = 14) a.u.; *P* < 0.0001. Red autofluorescence data are presented as mean/maximum values ± s.d.; *n* represents the number of biologically independent animals. Two-tailed *t*-test was used to determine the statistical significance. ***P < 0.0001. DIC, differential interference contrast. **d**, Mating induces breakdown of intestinal barrier function. Left, representative images of Day 4 mated and unmated *fog-2* hermaphrodites after soaking in blue dye overnight; right, quantification of intestinal leakage in the population of mated and unmated worms. **e**, Experimental design of germline isolation from mated and unmated *fog-2* hermaphrodites. **f**, Left, over 8,000 mRNAs express at a lower level in the distal germline compared with whole animals (regraphed using published data^35^). Right, expression levels in mated and unmated germlines (mRNA-seq data from this study) for the same set of mRNAs as on the left. Axis unit, log_10_(RPM). **g**, PCA of normalized read counts from the mRNA transcriptomes of the isolated germlines. **h**, Volcano plot of mRNA-seq transcriptome data displaying the pattern of gene expression values for mated versus unmated germlines. Significantly differentially-expressed genes (adjusted *P* value (*P*_adj_) ≤ 0.05) are highlighted in red (upregulation) and blue (downregulation), whereas genes with no significant expressional changes are in gray. The pie chart (right) summarizes the number of significantly differentially-expressed genes in the mated germline compared with the unmated germline. *P* values are calculated in DESeq2 using the Wald test. Full list of genes for **h** is available as Source Data. **i**, Tissue enrichment analysis of significantly differentially-expressed genes in the mated germline (https://wormbase.org/tools/enrichment/tea/tea.cgi). **j**,**k**, GO enrichment analysis of significantly up- (**j**) and down- (**k**) regulated genes in the mated germline (https://wormbase.org/tools/enrichment/tea/tea.cgi). *P* values in **i**–**k** were calculated by a hypergeometric function after FDR correction using a Benjamini–Hochberg algorithm as previously described^86^. Source data

**Extended Data Fig. 1:**
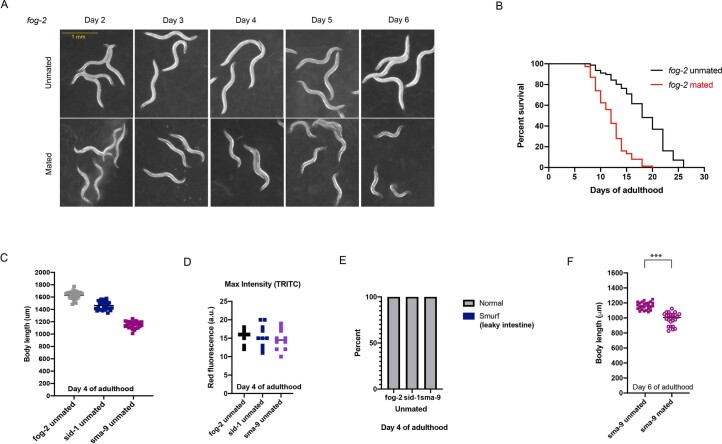
Mating induces shrinking and other aging phenotypes that are independent of developmental body size regulation. A) Representative pictures of the unmated and mated hermaphrodites from Day 2 – Day 6 of adulthood. Mating occurs on Day 1 for 24 hours. B) Representative lifespan of unmated and mated *fog-2* hermaphrodites. Unmated: 18.2±0.6 days, n = 85; mated: 12.0±0.3 days, n = 83, p < 0.001. Lifespan results are presented as mean values ± std error for all the lifespan assays in this study. n represents the number of biologically independent animals in the lifespan assay. Significance was determined by Log-rank (Mantel–Cox) test for lifespan comparisons. C) Quantification of unmated Day 4 worms with various body sizes (wildtype *fog-2*, *sid-1*, and *sma-9*). Unmated *fog-2*: 1630±68 μm (n = 25), unmated *sid-1*: 1462±72 μm (n = 25), unmated *sma-9*: 1151±58 μm (n = 25). D) Quantification of maximum red autofluorescence of unmated wildtype *fog-2*, *sid-1*, and *sma-9* worms. No difference was observed despite their different body sizes. *fog-2*: 15.4±1.9 a.u. (n = 10), *sid-1*: 15.6±3.1 a.u. (n = 10), *sma-9*: 14.6±2.8 a.u. (n = 10). Red autofluorescence data are presented as maximum values ± SD. n represents the number of biologically independent animals. E) Quantification of intestinal leakage of these unmated Day 4 wildtype *fog-2*, *sid-1*, and *sma-9* hermaphrodites. 50 worms were scored for each genotype. F) Body size measurement of mated and unmated *sma-9(wk55)* mutants. Day 6 unmated *sma-9*: 1160±49 μm (n = 25), mated *sma-9*: 989±82 μm (n = 25), *** p < 0.0001.

Mating causes shrinking and decreased lifespan^12^. Day 1 adult self-spermless hermaphrodites (*fog-2*) mated for 24 h with Day 1 adult males live 40% shorter than their unmated counterparts, and shrink by up to 30%^12^ (Fig. 1a,b and Extended Data Fig. 1a,b). (*fog-2* hermaphrodites, the control genotype, are self-spermless; successful mating can be determined quickly by the presence of fertilized eggs inside the worm.) In addition to shrinking, mated animals also display an acceleration in age-related autofluorescence^31^ and intestinal barrier dysfunction (Fig. 1c,d); this phenomenon is specific to mating, as developmentally small *sma-9* mutants do not exhibit these defects (Extended Data Fig. 1c–e) and still shrink upon mating (Extended Data Fig. 1f)^12^. The longevity decrease after mating is mediated by multiple factors, including seminal fluid transfer, male sperm-induced germline hyperactivity, and male pheromone toxicity^12,13,32–34^. While seminal fluid-mediated death involves insulin-like peptides and the insulin/FOXO signaling pathway, the germline-specific pro-aging signal that induces shrinking is unknown^12^.

To identify the germline pro-aging signal that induces postmating physiological changes and early death, we dissected distal germlines from mated and unmated hermaphrodites, and performed mRNA sequencing (mRNA-seq) and small RNA sequencing (RNA-seq) (Fig. 1e). Hermaphrodites were mated with young males from Day 1 of adulthood for 2 days before germline dissection. Because mating causes a significant reduction of the mitotic distal germline region (about 30% decrease in size and 40% decrease in nuclei number^12^), we used an equal amount of RNA, rather than an equal number of worms or germline, from each germline sample to make our sequencing libraries, and sequencing reads were normalized in our analysis. In unmated hermaphrodites, over 8,000 mRNAs were previously reported to have reduced expression in the distal germline compared with whole animals^35^ (Fig. 1f, left). These distal germline-depleted genes in unmated worms were not enriched in the mated germline (Fig. 1f, right). Therefore, the significantly differentially-expressed genes we identified here are induced specifically by mating rather than by reduction in size of the mated germline.

A total of 666 genes were significantly differentially upregulated and 590 were downregulated in mated germlines compared with unmated germlines (Fig. 1g,h and Source Data). These genes were enriched in germline, reproductive system and gonadal primordium function, confirming successful dissection of the germline with minimum contamination by other tissues (Fig. 1i). Upregulated mated germline genes were enriched for ribosome, cuticle, protein heterodimerization and peptide biosynthesis (Fig. 1j), which are essential for rapid germ cell proliferation and cellularization. One of the most upregulated genes, *sri-40* (Fig. 1h), was also significantly induced in whole body after mating with males and, when reduced, extends lifespan with and without males, suggesting its involvement in general protection of mating-induced death^34^. Genes downregulated in the mated germline include metabolic processes (rRNA, cellular aromatic compound and heterocycle metabolic process) (Fig. 1k), indicating a shift of metabolic activities in the mated germline.

### piRNAs are downregulated in the mated germline

**Fig. 2:**
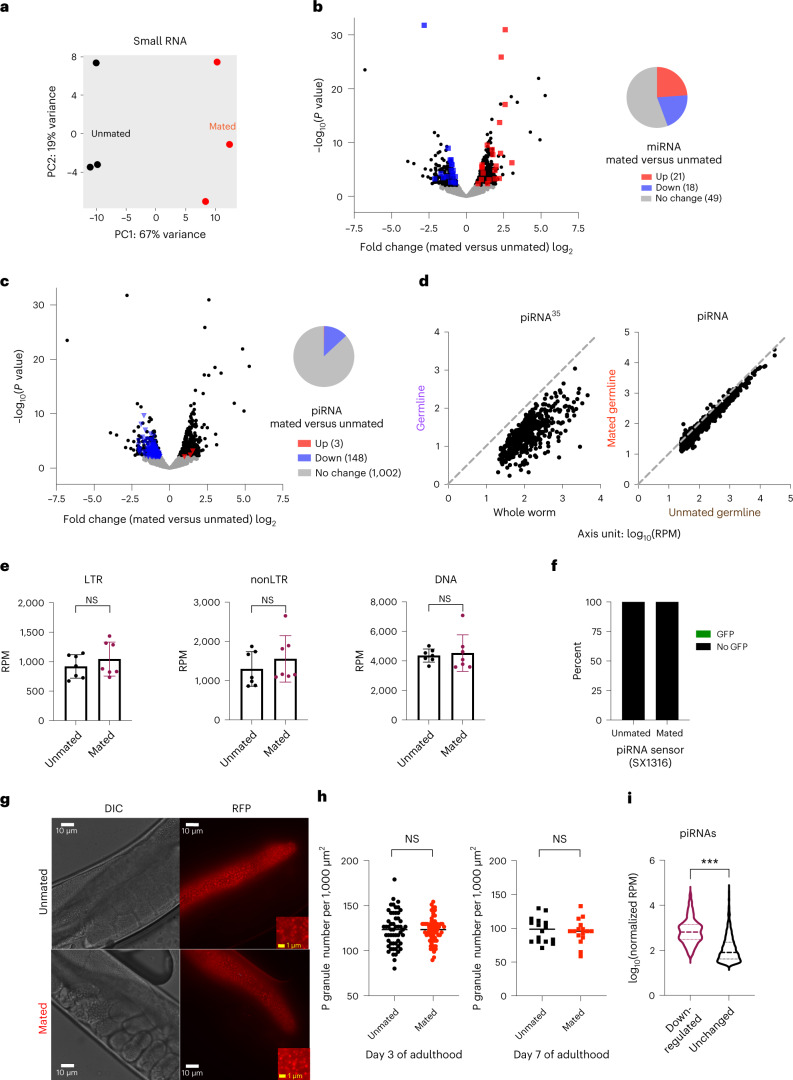
Mating downregulates a small and specific set of piRNAs without a general disruption of the piRNA pathway. **a**, PCA of normalized read counts from the small RNA transcriptomes of the isolated germlines. **b**,**c**, Volcano plots of small RNA-seq transcriptome data of mated versus unmated germlines. Black dots, significantly differentially-expressed small RNAs (full list is available as Source Data); gray dots, small RNAs with no significant expression changes after mating. Significantly differentially-expressed miRNAs (**b**) or piRNAs (**c**) (*P*_adj_ ≤ 0.05) are highlighted in red (upregulation) and blue (downregulation). The pie chart (right) summarizes the number of significantly differentially-expressed and unchanged miRNAs (**b**) or piRNAs (**c**) in the mated germline compared with the unmated germline. **d**, Left, over 1,000 piRNAs express at a lower level in the distal germline compared with whole animals (regraphed using published data^35^). Right, expression levels in mated and unmated germlines (small RNA-seq data from this study) for the same set of piRNAs as on the left. Axis unit, log_10_(RPM). **e**, Mating does not change levels of main types of TE (Source Data). Seven biological replicates in total. LTR (left) unmated, 919 ± 200 RPM; mated, 1,044 ± 289 RPM; *P* = 0.3624; nonLTR (middle) unmated, 1,299 ± 444 RPM; mated, 1,556 ± 593 RPM; *P* = 0.3761; DNA (right) unmated, 4,360 ± 445 RPM; mated, 4,527 ± 1,240 RPM; *P* = 0.7432. Expression levels are presented as mean values ± s.d. Two-tailed *t*-test was used to determine statistical significance. NS, no statistical difference. **f**, The general piRNA sensor (SX1316) remains silenced in mated worms; 25 worms were checked for each condition. **g**,**h**, Mating does not affect the morphology or density of P granules. **g**, Representative images of mated and unmated Day 3 YY968 worms (P granules labeled by PGL-1::RFP). Mating started on Day 1 for 2 days. **h**, Quantification of P granule density (blindly scored) of Day 3 (left) and Day 7 (right) worms: Day 3 unmated, 123 ± 20 per 1,000 μm^2^ (*n* = 55); mated, 123 ± 14 per 1,000 μm^2^ (*n* = 63); *P* = 0.9363. Day 7 unmated, 97 ± 18 per 1,000 μm^2^ (*n* = 16); mated, 95 ± 18 per 1,000 μm^2^ (*n* = 16); *P* = 0.7217. Data are presented as mean values ± s.d.; *n*, number of biologically independent germlines. Two-tailed unpaired *t*-test was used to determine statistical significance. **i**, Mating-induced downregulated piRNAs are expressed at a significantly higher level in the unmated germline compared with the other over 1,000 unchanged piRNAs. ***P < 0.0001; two-tailed unpaired *t*-test was used to determine statistical significance. Source data

Several species of small RNAs are expressed primarily in the germline and regulate germline function. For example, microRNAs are critical in lifespan regulation after germline removal^27,36^. Endogenous siRNAs are required for maintenance of proteostasis and lifespan extension in germlineless worms^37^, and play critical roles in shaping the germline transcriptome^35^. PIWI-associated RNAs (piRNAs) are expressed predominantly in the germline and are required to maintain germline integrity and fertility^38^. Differential expression analysis of small RNAs from dissected mated and unmated germlines revealed 440 significantly upregulated and 296 downregulated small RNAs in the mated germline (Fig. 2a and Source Data). Whereas miRNAs showed equal distribution in up- and downregulation (Fig. 2b), a small set of piRNAs were almost exclusively (98%) downregulated in the mated germline (148 downregulated versus 3 upregulated; Fig. 2c), indicating that mating triggers a unique response in germline piRNA abundance. While many species of small RNAs, including over 1,500 piRNAs, are depleted in the distal germline compared with the whole worm in unmated hermaphrodites^35^ (Fig. 2d, left), we do not see a general bias in piRNA expression; that is, these piRNAs were not enriched in the mated germline (Fig. 2d, right), suggesting that mating-induced downregulation of specific piRNAs is not due simply to the reduction of the mitotic germline region caused by mating.

The best-characterized function of piRNAs is the silencing of transposable elements (TE) to preserve genome integrity^39,40^. We observed that the main classes of TE were expressed at similar levels in unmated and mated germlines (Fig. 2e and Source Data), suggesting that piRNA changes are unlikely to be regulating TE in this context. In addition, mated and unmated worms showed no difference in activity of a general piRNA sensor, that is, desilenced if the piRNA pathway is completely nonfunctional^41,42^ (Fig. 2f). P granules are essential for piRNA-mediated gene silencing^43^; however, neither P granule density nor morphology was changed immediately after mating (Day 3) or when shrinking is obvious (Day 7) (Fig. 2g,h), suggesting that general piRNA function in P granules is not affected by mating. Moreover, although we detected more than 1,000 piRNAs in our dissected germlines that were unchanged upon mating (Fig. 2c), these specific 148 mating-induced downregulated piRNAs were expressed at a significantly higher levels in the unmated germline compared with the other over 1,000 unchanged piRNAs (Fig. 2i). Together, our data suggest that mating leads to the downregulation of a small and specific set of piRNAs without a general disruption of the piRNA pathway.

### Mating-induced changes require the germline piRNA pathway

**Fig. 3:**
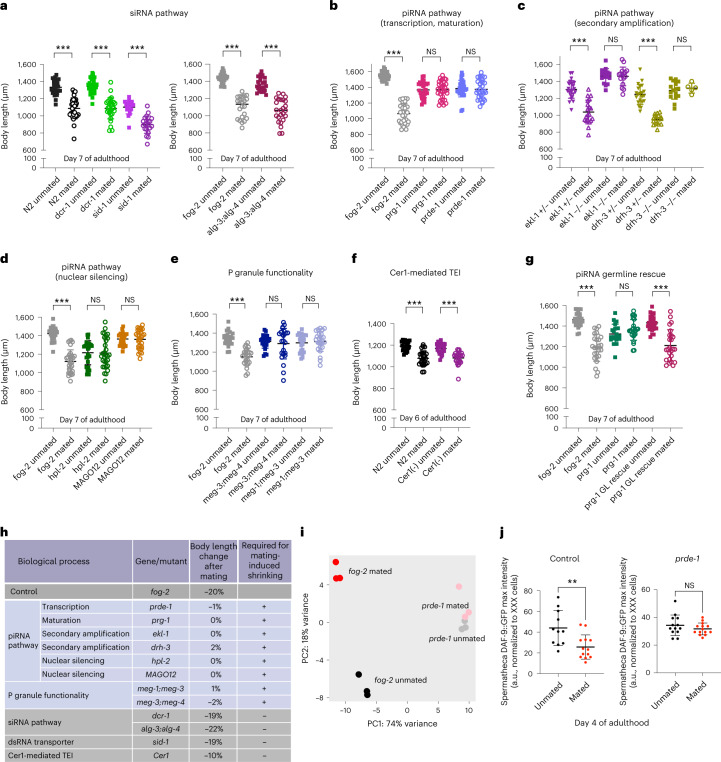
Mating-induced shrinking and transcriptional changes require the functional piRNA pathway in the germline. **a**–**f**, Body size measurements of mated and unmated hermaphrodites related to different small RNA pathways. **a**, Mutants that are defective in miRNA or siRNA pathways still shrink after mating. N2 unmated, 1,334 ± 81 μm (*n* = 30); N2 mated, 1,088 ± 122 μm (*n* = 31); ***P < 0.001. *dcr-1(mg375)* unmated, 1,347 ± 84 μm (*n* = 30); mated, 1,088 ± 122 μm (*n* = 30); ***P < 0.001. *sid-1(qt9)* unmated, 1,102 ± 78 μm (*n* = 20); mated, 898 ± 101 μm (*n* = 21); ***P < 0.001. *fog-2(q71)* unmated, 1,436 ± 54 μm (*n* = 30); mated, 1,099 ± 128 μm (*n* = 22); ***P < 0.001. *alg-4(ok1041); alg-3(tm1155)* unmated, 1,364 ± 78 μm (*n* = 25); mated, 1,060 ± 136 μm (*n* = 25); ***P < 0.001. **b**–**f**, Mutants in which multiple steps of the piRNA pathway are affected are resistant to mating-induced shrinking. **b**, piRNA pathway mutants do not shrink after mating. *fog-2(q71)* unmated, 1,546 ± 55 μm (*n* = 30); mated, 1,065 ± 127 μm (*n* = 30); ***P < 0.001. *prg-1(n4357)* unmated, 1,368 ± 100 μm (*n* = 30); mated, 1,369 ± 114 μm (*n* = 30); *P* = 0.9603. *prde-1(mj207)* unmated, 1,385 ± 106 μm (*n* = 30); mated, 1,376 ± 120 μm (*n* = 31); *P* = 0.7581. **c**, *ekl-1* and *drh-3* mutant alleles are maintained by chromosome balancers. Postmating body size of siblings with homozygous mutant alleles (–/–, no balancer) and heterozygous mutant allele (+/–, with the balancer) was measured in the same experiment. *ekl-1(ok1197)[+/–]* unmated, 1,306 ± 105 μm (*n* = 25); mated, 1,039 ± 140 μm (*n* = 20); ***P < 0.001. *ekl-1(ok1197)[–/–]* unmated, 1,464 ± 87 μm (*n* = 16); mated, 1,460 ± 111 μm (*n* = 16); *P* = 0.9063. *drh-3(tm1217)[+/–]* unmated, 1,247 ± 117 μm (*n* = 21); mated, 951 ± 58 μm (*n* = 16); ***P < 0.001. *drh-3(tm1217)[–/–]* unmated, 1,294 ± 104 μm (*n* = 16); mated, 1,321 ± 58 μm (*n* = 5); *P* = 0.5863. **d**, *fog-2(q71)* unmated, 1,422 ± 69 μm (*n* = 30); mated, 1,122 ± 125 μm (*n* = 30); ***P < 0.001. *hpl-2(ok916)* unmated, 1,215 ± 130 μm (*n* = 30); mated, 1,212 ± 164 μm (*n* = 30); *P* = 0.9286. MAGO12 unmated, 1,365 ± 65 μm (*n* = 30); mated, 1,361 ± 100 μm (*n* = 30); *P* = 0.8577. **e**, *fog-2(q71)* unmated, 1,349 ± 73 μm (*n* = 25); mated, 1,151 ± 89 μm (*n* = 25); ***P < 0.001. *meg-3(tm4259) meg-4(ax2026)* unmated, 1,312 ± 71 μm (*n* = 25); mated, 1,289 ± 161 μm (*n* = 25); *P* = 0.5179. *meg-1(vr10) meg-3(tm4259)* unmated, 1,298 ± 89 μm (*n* = 25); mated, 1,311 ± 104 μm (*n* = 25); *P* = 0.6330. **f**, N2 unmated, 1,191 ± 40 μm (*n* = 25); mated, 1,078 ± 69 μm (*n* = 25); ***P < 0.001. *Cer1*(–) (RNAi for three generations) unmated, 1,170 ± 46 μm (*n* = 25); mated, 1,081 ± 59 μm (*n* = 25); ***P < 0.001. TEI, transgenerational epigenetic inheritance. **g**, Mating induces shrinking in hermaphrodites with germline-specific rescue of the piRNA pathway. *fog-2(q71)* unmated, 1,462 ± 68 μm (*n* = 25); mated, 1,189 ± 131 μm (*n* = 25); ***P < 0.001. *prg-1(n4357)* unmated, 1,322 ± 96 μm (*n* = 20); mated, 1,363 ± 101 μm (*n* = 25); *P* = 0.1803. Germline-specific *prg-1* rescue (CQ655) unmated, 1,431 ± 74 μm (*n* = 25); mated, 1,212 ± 152 μm (*n* = 25); ***P < 0.001. **h**, Summary of the genetic requirement for mating-induced shrinking. **i**, PCA of normalized read counts from the mRNA transcriptomes of the isolated germlines. Black, *fog-2* unmated; red, *fog-2* mated; gray, *prde-1* unmated; pink, *prde-1* mated. Full list of mating-induced significantly differentially-expressed genes in *prde-1* germline is available as Source Data. **j**, Quantification of maximum value of *Pdaf-9::daf-9::gfp* expression in the spermatheca in wildtype (left) and *prde-1* (right) background. All measurements were normalized to the DAF-9 expression in the XXX cells in the head, which is not affected by mating. Unmated control, 44.1 ± 17.0 a.u. (*n* = 11); mated control, 25.7 ± 11.7 a.u. (*n* = 13); *P* = 0.0049; unmated *prde-1*, 34.4 ± 7.4 a.u. (*n* = 13); mated *prde-1*, 31.7 ± 4.2 a.u. (*n* = 14); *P* = 0.2676. Data are presented as maximum values ± s.d.; *n*, number of biologically independent animals. Two-tailed *t*-test was used to determine statistical significance. **P < 0.01. Source data

**Extended Data Fig. 2:**
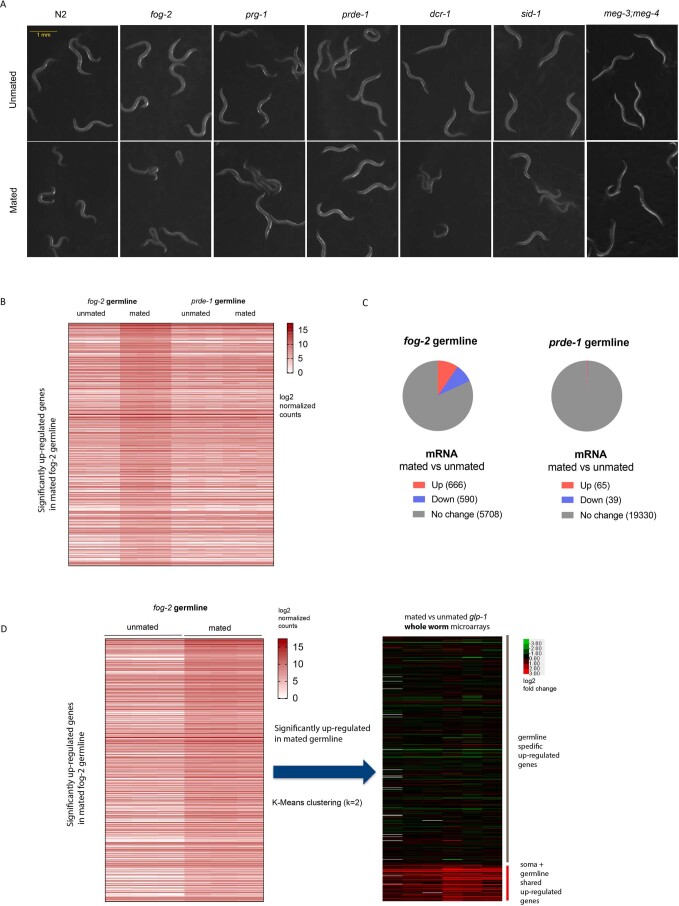
piRNA pathway mutants are resistant to mating-induced shrinking and lack transcriptional responses in the germline. A) Representative pictures of the unmated and mated hermaphrodites related to different small RNA pathways on Day 6/7 of adulthood. At least 20-30 worms (n) were measured for each condition and genotype. See Fig. 3A (N2, *dcr-1*, *sid-1*), Fig. 3B (*fog-2*, *prg-1*, *prde-1*), Fig. 3E(*meg-3;meg-4*) for comparisons and statistics. B) Heatmap of genes that are significantly up-regulated in mated *fog-2* germline. The data are displayed as log2(normalized counts). Mating-induced up-regulation in expression is completely abolished in piRNA pathway-deficient *prde-1* germlines. C) Pie chart summary of mating-induced expressional changes in normal functional *fog-2* germline (left) and piRNA pathway deficient *prde-1* germline (right). D) K-Means (k = 2) clustering separates the significantly up-regulated genes in *fog-2* mated germline to germline-specific transcriptional response and generic transcriptional response to mating.

To further test which types of small RNA are required for germline-specific mating-induced response, we measured body size changes after mating for different types of small RNA pathway mutants, as shrinking serves as a phenotypic readout for the germline-emanating pro-aging signal^12^. We mated mutant hermaphrodites with young males for 2 days starting on Day 1 of adulthood and measured their body size on Day 6/7 of adulthood (Extended Data Fig. 2a). DCR-1 is required for both miRNA and siRNA processing^44–46^. Like wildtype animals, after mating, *dcr-1* hermaphrodites still shrank (Fig. 3a and Extended Data Fig. 2a), suggesting that Dicer function is unnecessary for the germline-to-soma shrinking signal. ALG-3 and ALG-4 are Argonaute proteins that bind and stabilize 26G siRNAs in the germline and mediate their effector functions^47,48^; alg-3;alg-4 double mutants still shrank after mating (Fig. 3a). Likewise, mutants of the dsRNA transporter SID-1 were also susceptible to mating-induced shrinking (Fig. 3a), suggesting that mating-induced shrinking does not rely on functional endogenous small RNA pathways.

Next, we tested mutants for factors in the piRNA pathway, including piRNA transcription (*prde-1* (ref. 49)), maturation (*prg-1* (refs. 50–52), secondary amplification (*ekl-1*, *drh-3* (refs. 41, 53)), and nuclear silencing (*hpl-2* (ref. 54)), as well as MAGO12, which has loss-of-function mutations in multiple Argonaute protein-encoding genes^53^. Unlike the mutants involved in siRNA processing (Fig. 3a), none of these piRNA pathway mutants shrank after mating (Fig. 3b–d and Extended Data Fig. 2a), indicating that the piRNA pathway is required for mating-induced shrinking in the hermaphrodites. Moreover, one genomic copy of the secondary amplification genes *ekl-1* and *drh-3* restored the susceptibility of worms to mating-induced shrinking (indicated by ‘+/–’; Fig. 3c). Together, our data suggest that piRNA pathway function is critical for the signal that conveys the mated state of the germline to the soma.

piRNAs scan most of the transcriptome while located in P granules (*Caenorhabditis elegans* germ granules)^43,55,56^, and disruption of P granules compromises piRNA-mediated silencing^43^. P granule distribution and assembly/disassembly dynamics depend on maternal-effect germline (MEG) proteins^57^. We found that *meg* double mutants in which P granule functionality is severely impaired^57^ were also resistant to mating-induced shrinking (Fig. 3e), demonstrating again that the germline-mediated postmating shrinking requires a functional piRNA pathway and P granules.

We recently found that the *Cer1* retrotransposon is required for communication between the germline and neurons in the transgenerational inheritance of pathogen avoidance, which also requires the germline and piRNAs^58^. However, worms lacking *Cer1* retrotransposon-encoded capsids still shrank after mating (Fig. 3f), suggesting that *Cer1* is not involved in mating-induced shrinking.

Although piRNAs are expressed predominantly in the germline, the piRNA pathway also functions in somatic tissue^59^. To determine whether the piRNA pathway is required in the germline to mediate mating-induced shrinking, we tested a germline-specific *prg-1* rescue strain; mating induced shrinking in these animals (Fig. 3g), confirming that piRNA function in the germline is sufficient to mediate postmating shrinking (Fig. 3h).

Next, we wondered whether the piRNA pathway is required for the dramatic mRNA transcriptional changes in mated germlines. To address this question, we isolated mated and unmated distal germlines from *prde-1* mutants, which are defective in piRNA biosynthesis^49^, and performed mRNA-seq. Principal component analysis (PCA) revealed that the lack of a functional piRNA pathway in *prde-1* worms eliminated most germline transcriptional differences induced by mating (Source Data, Fig. 3i and Extended Data Fig. 2b,c). *Prde-1* mutation eliminated the upregulation of genes in mated germlines (Extended Data Fig. 2b), indicating that the mating-induced transcriptional changes in the germline are also dependent on a functional piRNA pathway.

We previously found that *daf-9* and *daf-12* are required for mating-induced shrinking and that DAF-9::GFP is reduced after mating in spermatheca^12^. However, in the *prde-1* background, we did not observe mating-induced reduction of DAF-9::GFP signal, suggesting that *prde-1* and piRNA regulation lie upstream of the spermatheca and *daf-9* regulation (Fig. 3j).

### Hedgehog signaling may be the mysterious pro-aging signal

Both postmating shrinking and transcriptional changes require piRNA pathway function, implying that the germline pro-aging signal must be related to piRNAs. Since the pro-aging signal is amplified in mated germlines, but specific piRNAs are downregulated, piRNAs themselves are unlikely to be the direct signal. Nor did we observe expression differences in TE between unmated and mated germlines (Fig. 2e), suggesting that piRNA regulation of TE is unlikely to be the signal. Therefore, we hypothesized that the mating-induced pro-aging process may be regulated through another main role of piRNAs, endogenous gene silencing^60^. Mating-induced downregulation of piRNAs may release the silencing effect on specific piRNA targets, upregulating the expression of these genes, making piRNAs’ targeted genes more likely candidates for the germline pro-aging signal.

**Fig. 4:**
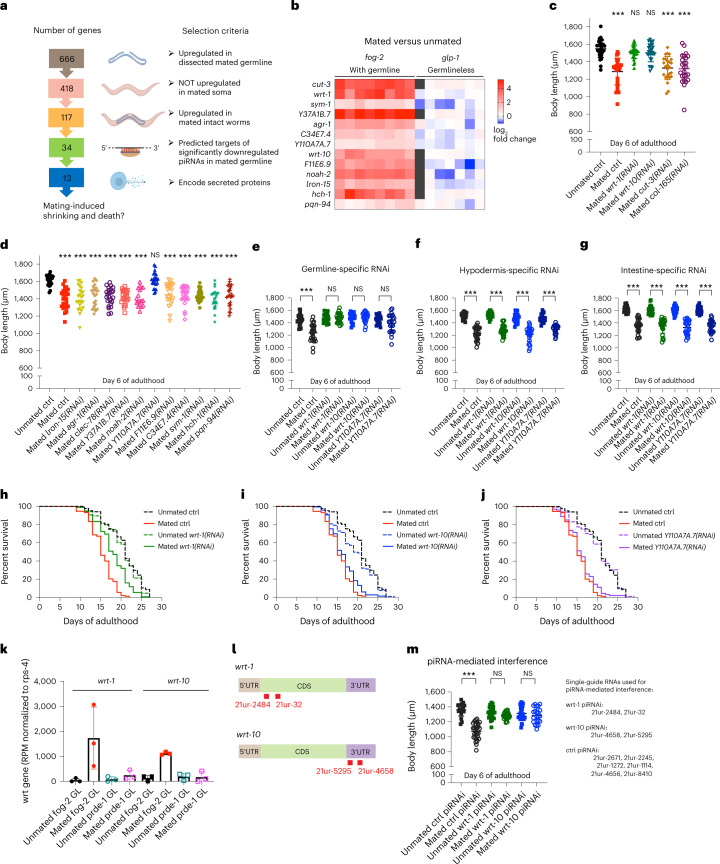
Secreted Hedgehog-like ligands in the germline are required for mating-induced shrinking and early death. **a**, Scheme describing the strategy and criteria to narrow the list of genes (Source Data) that might encode the germline pro-aging signal. **b**, Heatmap of the final 13 germline pro-aging signal candidates. Their mating-induced upregulation is completely lost in mated germlineless worms. The data are displayed as log_2_(fold change) of expression level in mated versus unmated whole worms. Left, *fog-2* worms with functional germlines; right, *glp-1* germlineless worms. **c**,**d**, Body size measurements of mated worms with RNAi treatment of individual candidate genes. **c**, *fog-2(q71)* unmated (control (ctrl) RNAi), 1,549 ± 99 μm (*n* = 30); mated (control RNAi), 1,288 ± 155 μm (*n* = 25); ***P < 0.001; mated *wrt-1(RNAi)*, 1,511 ± 72 μm (*n* = 27); *P* = 0.1070; mated *wrt-10(RNAi)*, 1,503 ± 103 μm (*n* = 28); *P* = 0.0917; mated *cut-3(RNAi)*, 1,329 ± 133 μm (*n* = 25); ***P < 0.001; mated *col-165(RNAi)*, 1,321 ± 165 μm (*n* = 25); ***P < 0.001. **d**, *fog-2(q71)* unmated(control RNAi), 1,615 ± 58 μm (*n* = 25); mated (control RNAi), 1,404 ± 116 μm (*n* = 25); ***P < 0.001; mated *lron-15(RNAi)*, 1,405 ± 133 μm (*n* = 25); ***P < 0.001; mated *agr-1(RNAi)*, 1,433 ± 124 μm (*n* = 25); ***P < 0.001; mated *clec-78(RNAi)*, 1,425 ± 101 μm (*n* = 25); ***P < 0.001; mated *Y37A1B.7(RNAi)*, 1,421 ± 78 μm (*n* = 25); ***P < 0.001; mated *noah-2(RNAi)*, 1,414 ± 100 μm (*n* = 25); ***P < 0.001; mated *Y110A7A.7(RNAi)*, 1,615 ± 85 μm (*n* = 25); *P* = 0.9780; mated *F11E6.9(RNAi)*, 1,461 ± 124 μm (*n* = 25); ***P < 0.001; mated *C34E7.4(RNAi)*, 1,441 ± 106 μm (*n* = 25); ***P < 0.001; mated *sym-1(RNAi)*, 1,441 ± 66 μm (*n* = 25), ***P < 0.001; mated *hch-1(RNAi)*, 1,401 ± 109 μm (*n* = 25); ***P < 0.001; mated *pqn-94(RNAi)*, 1,433 ± 118 μm (*n* = 25); ***P < 0.001. **e**–**g**, Germline-specific knockdown (**e**) but not hypodermis-specific (**f**) or intestine-specific (**g**) knockdown of *wrt-1*, *wrt-10* and *Y110A7A*.7 protects the mated hermaphrodites from shrinking. **e**, Germline-specific RNAi strain (DCL569) unmated (control RNAi), 1,449 ± 89 μm (*n* = 25); mated (control RNAi), 1,239 ± 141 μm (*n* = 25); ***P < 0.001; unmated *wrt-1(RNAi)*, 1,477 ± 71 μm (*n* = 25); mated *wrt-1(RNAi)*, 1,479 ± 74 μm (*n* = 25); *P* = 0.9211; unmated *wrt-10(RNAi)*, 1,485 ± 76 μm (*n* = 25); mated *wrt-10(RNAi)*, 1,491 ± 77 μm (*n* = 25); *P* = 0.7701; unmated *Y110A7A.7(RNAi)*, 1,441 ± 66 μm (*n* = 20); mated *Y110A7A.7(RNAi)*, 1,391 ± 147 μm (*n* = 23); *P* = 0.1698. **f**, Hypodermis-specific RNAi strain (CQ479) unmated (control RNAi), 1,511 ± 51 μm (*n* = 22); mated (control RNAi), 1,230 ± 105 μm (*n* = 26); ***P < 0.001; unmated *wrt-1(RNAi)*, 1,528 ± 49 μm (*n* = 25); mated *wrt-1(RNAi)*, 1,282 ± 83 μm (*n* = 25); ***P < 0.001; unmated *wrt-10(RNAi)*, 1,477 ± 75 μm (*n* = 21); mated *wrt-10(RNAi)*, 1,218 ± 120 μm (*n* = 26); ***P < 0.001; unmated *Y110A7A.7(RNAi)*, 1,474 ± 67 μm (*n* = 17); mated *Y110A7A.7(RNAi)*, 1,297 ± 67 μm (*n* = 24); ***P < 0.001. **g**, Intestine-specific RNAi strain (IG1839) unmated (control RNAi), 1,627 ± 53 μm (*n* = 25); mated (control RNAi), 1,357 ± 111 μm (*n* = 25); ***P < 0.001; unmated *wrt-1(RNAi)*, 1,603 ± 67 μm (*n* = 25); mated *wrt-1(RNAi)*, 1,352 ± 118 μm (*n* = 25); ***P < 0.001; unmated *wrt-10(RNAi)*, 1,605 ± 58 μm (*n* = 25); mated *wrt-10(RNAi)*, 1,326 ± 110 μm (*n* = 25); ***P < 0.001; unmated *Y110A7A.7(RNAi)*, 1,607 ± 52 μm (*n* = 25); mated *Y110A7A.7(RNAi)*, 1,348 ± 96 μm (*n* = 25); ***P < 0.001. **h**–**j**, RNAi knockdown of *wrt-1* and, to a lesser degree, *wrt-10* increases the lifespan specifically of mated worms. Mated control, 15.3 ± 0.2 days, *n* = 199; unmated control, 20.7 ± 0.5 days, *n* = 141. **h**, Unmated *wrt-1(RNAi)*, 20.2 ± 0.4 days, *n* = 180; mated *wrt-1(RNAi)*, 18.0 ± 0.3 days, *n* = 190. Log-rank (Mantel–Cox) test revealed that the lifespan of mated *wrt-1(RNAi)* worms was significantly different from that of mated control worms (***P < 0.001). Two-way ANOVA revealed that mating-induced lifespan decrease of *wrt-1(RNAi)* worms was significantly different from that of control worms (*P* = 0.0016). **i**, Unmated *wrt-10(RNAi)*, 19.3 ± 0.4 days, *n* = 237; mated *wrt-10(RNAi)*, 16.4 ± 0.3 days, *n* = 203. Log-rank (Mantel–Cox) test revealed that the lifespan of mated *wrt-10(RNAi)* worms was significantly different from that of mated control worms (*P* = 0.0214). However, two-way ANOVA revealed that mating-induced lifespan decrease of *wrt-10(RNAi)* worms was not significantly different from that of control worms (*P* = 0.6789). **j**, Unmated *Y110A7A.7(RNAi)*, 18.8 ± 0.5 days, *n* = 196; mated *Y110A7A.7(RNAi)*, 15.8 ± 0.2 days, *n* = 233. Log-rank (Mantel–Cox) test revealed that the lifespan of mated *Y110A7A.7(RNAi)* worms was not significantly different from that of mated control worms (*P* = 0.0709). Two-way ANOVA revealed that mating-induced lifespan decrease of *Y110A7A.7(RNAi*) worms was not significantly different from that of control worms (*P* = 0.0509). **k**, Expression levels of *wrt-1* and *wrt-10* genes in the germline of *fog-2* and *prde-1* worms (three biological replicates in total, each replicate contains a pool of 200 dissected germlines). Read counts were normalized to the housekeeping gene *rps-4* in each sample. Expression data are presented as mean values ± s.d. Expression level of *wrt-1* in unmated *fog-2* germline: 44 ± 76 RPM; mated *fog-2* germline, 1,732 ± 1,242 RPM; unmated *prde-1* germline: 84 ± 96 RPM; mated *prde-1* germline, 246 ± 188 RPM. Expression level of *wrt-10* in unmated *fog-2* germline: 132 ± 114 RPM; mated *fog-2* germline, 1,118 ± 62 RPM; unmated *prde-1* germline, 189 ± 137 RPM; mated *prde-1* germline, 170 ± 212 RPM. **l**, piRNAs targeting *wrt-1* and *wrt-10* target different regions. **m**, Overexpressing *wrt-1-* or *wrt-10*-targeting piRNAs is sufficient to prevent mating-induced shrinking. Body size measurements: control (overexpressing 21ur-2671, 21ur-2245, 21ur-1272, 21ur-1114, 21ur-4656 and 21ur-8410) unmated, 1,361 ± 84 μm (*n* = 20); mated, 1,073 ± 109 μm (*n* = 28); ***P < 0.001. *Wrt-1*-targeting piRNAi (overexpressing 21ur-2484 and 21ur-32) unmated, 1,320 ± 82 μm (*n* = 25); mated, 1,283 ± 47 μm (*n* = 26); *P* = 0.0616. *Wrt-10*-targeting piRNAi (overexpressing 21ur-4658 and 21ur-5295) unmated, 1,312 ± 97 μm (*n* = 25); mated, 1,284 ± 102 μm (*n* = 22); *P* = 0.3427. Source data

Reasoning that the pro-aging signal should be amplified after mating, we started with the list of 666 genes that are significantly upregulated in the mated germlines we identified in our mRNA-seq analysis (Fig. 4a). Since the pro-aging signal originates from the germline, it must be germline-specific; we subtracted those genes that are also upregulated in mated *glp-1* germlineless worms^34^ from the original list, reducing the number of candidates to 418 (Source Data, Fig. 4a and Extended Data Fig. 2d). Next, we reasoned that the amplified germline signal should be robust enough to be identified in the transcriptome of whole worms with an intact germline, since the germline accounts for a significant proportion of the whole worm biomass. We compared these 418 genes with our previous *fog-2* mated versus unmated whole-worm expression data^61^ and found that the two datasets share 117 genes (Supplementary Data 1). Furthermore, since piRNAs are required for mating-induced transcriptional changes and shrinking, the germline signal should also be dependent on piRNAs. Therefore, we compared the remaining 117 genes with potential predicted targets (using the default ‘relaxed’ piRNA targeting setting from piRTarBase^62^) of 148 significantly downregulated piRNAs identified in the mated germline (Supplementary Data 2); 34 of 117 genes were targets predicted to be regulated by the 148 piRNAs that were downregulated in mated germline (Supplementary Data 3). Finally, to relay the signal from the germline to the rest of the body, the gene product is likely to be secreted. We applied two prediction algorithms (Euk-mPLoc 2.0 (ref. 63) and SignalP^64^) to the 34 genes and found that 13 are predicted to encode secreted proteins. We then ranked the final 13 genes according to their expression fold change in the germline and in the whole worm, and their consistency in expression patterns across the samples (Fig. 4b and Supplementary Data 3).

**Extended Data Fig. 3:**
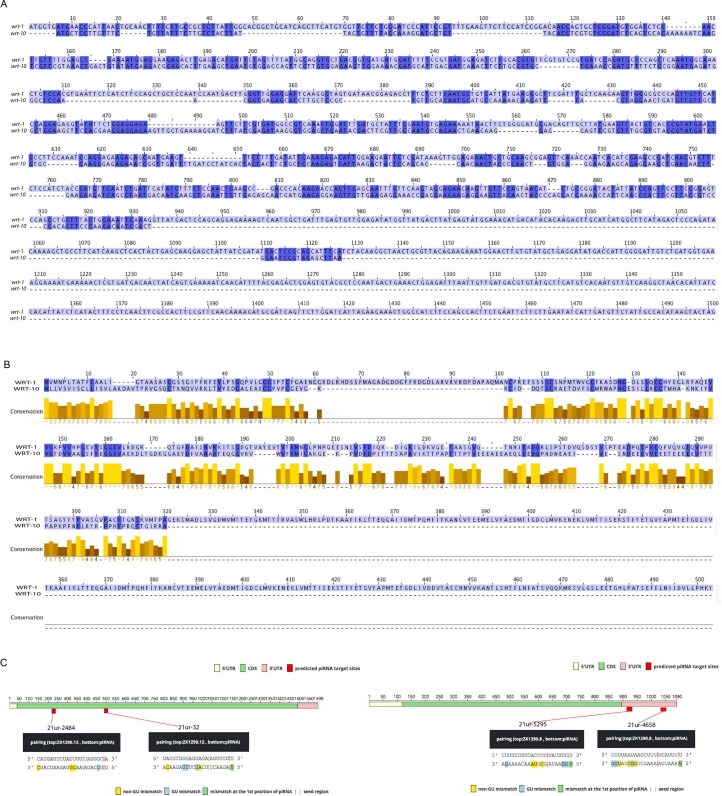
Sequence homology of *wrt-1* and *wrt-10*. A) Comparison of *wrt-1* and *wrt-10* cDNA sequences using Clustal Omega (https://www.ebi.ac.uk/Tools/msa/clustalo/). B) Comparison of WRT-1 and WRT-10 protein sequences using Clustal Omega. C) *wrt-1-* (left) and *wrt-10-* (right) targeting piRNAs: sequences and predicted binding sites. Figures were modified from piRTarBase.

To test whether any of these 13 genes might be the germline pro-aging signal, we measured body size postmating after RNAi treatment for each individual gene. Knockdown of three candidates prevented the mated worms from shrinking: two warthog (Hedgehog-like family) genes, *wrt-1* and *wrt-10*, and an uncharacterized gene, Y110A7A.7 (Fig. 4c, d and Extended Data Fig. 3a,b). Knocking down these three genes specifically in the germline (Fig. 4e), but not in the hypodermis (Fig. 4f) or the intestine (Fig. 4g), was sufficient to protect the mated hermaphrodites from shrinking, confirming that they are specifically required in the germline to mediate mating-induced shrinking, although *Y110A7A.7* reduction in the germline was less protective of mating-induced shrinking than was *wrt-1* or *wrt-10* knockdown.

**Extended Data Fig. 4:**
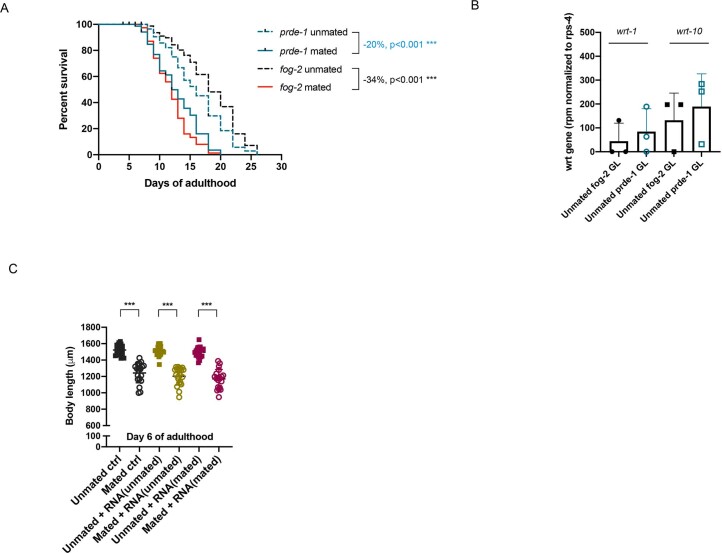
Inhibiting piRNA pathway and Hedgehog signaling largely ameliorates mating-induced early death. A) Lifespans of mated and unmated worms with defective piRNA pathway and control. *fog-2* unmated ctrl: 18.2±0.6 days, n = 85, *fog-2* mated ctrl: 12.0±0.3 days, n = 83, p < 0.001; *prde-1* unmated ctrl: 16.1±0.5 days, n = 89, *prde-1* mated ctrl: 12.8±0.5 days, n = 77, p < 0.001. Significance was determined by Log-rank (Mantel–Cox) test. B) Expression levels of *wrt-1* and *wrt-10* genes in the germline of unmated *fog-2* and *prde-1* worms (three biological replicates in total, each replicate contains a pool of 200 dissected germlines). Read counts were normalized to housekeeping gene *rps-4* in each sample. Expression data are presented as mean values ± SD. Expression level of *wrt-1* in unmated *fog-2* germline: 44±76 rpm (reads per million reads), unmated *prde-1* germline: 84±96 rpm. Expression level of *wrt-10* in unmated *fog-2* germline: 132±114 rpm, unmated *prde-1* germline: 189±137 rpm. C) Mating-induced shrinking is not affected by large amounts of extracted total RNA from unmated or mated worms. 2ug of total RNA of each type with RNase inhibitor was seeded on plate for the assay, Worms were transferred every two days onto freshly seeded plates with total RNA supplementation. Body size measurements: unmated control: 1524±66 μm (n = 19), mated control: 1242±119 μm (n = 21), ***p < 0.001; unmated with total RNA extracted from unmated whole worms: 1513±63 μm (n = 20), mated with total RNA extracted from unmated whole worms: 1202±107 μm (n = 20), ***p < 0.001; unmated with total RNA extracted from mated whole worms: 1496±64 μm (n = 20), mated with total RNA extracted from mated whole worms: 1170±115 μm (n = 20), ***p < 0.001.

In addition to preventing postmating shrinking, inhibiting the bona fide germline pro-aging signal should also increase the lifespan of mated worms. Consistent with this notion, reduction of *wrt-1* provided over 50% protection against mating-induced early death (significant effect determined by both log-rank test and two-way analysis of variance (ANOVA); Fig. 4h). Knocking down *wrt-10* yielded a much milder effect (10% protection, significant by log-rank test, *P* = 0.0214 but not significant by two-way ANOVA, *P* = 0.6789; Fig. 4i). By contrast, Y110A7A.7 RNAi did not prevent the worms from mating-induced death (Fig. 4j). Expression of both *wrt-1* and *wrt-10* genes is decreased in a mated *prde-1* mutant compared with mated *fog-2* animals (Fig. 4k). These results suggest that *wrt-1* and, to a much lesser extent, *wrt-10*, may play a critical role in mediating germline pro-aging signaling. Similarly, mating-induced death was partially attenuated in *prde-1* mutants, in which the mating-induced germline upregulation of *wrt-1* and *wrt-10* are abrogated (Extended Data Fig. 4a). The remaining unrescued lifespan decrease is probably caused by the germline-independent mating-induced death mechanisms downstream of seminal fluid transfer^12,14^.

### piRNAs targeting Hh ligands block mating-induced shrinking

To test the specificity of the piRNA pathway in regulating mating-induced shrinking, we overexpressed specific piRNAs in the germline via piRNA-mediated interference^65^. Among 148 significantly downregulated piRNAs in the mated germline, two piRNAs, 21ur-2484 and 21ur-32, are predicted to target *wrt-1*, whereas another two piRNAs, 21ur-4658 and 21ur-5295, are predicted to target *wrt-10* (piRTarBase; Fig. 4l and Extended Data Fig. 3c). *Wrt-1-* and *wrt-10-*targeting piRNAs were added to the synthetic piRNAi scaffold, while six other non-*wrt* gene targeting piRNAs that are also downregulated in mated germlines were used as the control. Overexpressing piRNAs targeting either *wrt-1* or *wrt-10* was sufficient to prevent mated worms from mating-induced shrinking (Fig. 4m). These data suggest that postmating shrinking—a pro-aging phenotype—is regulated directly by piRNAs that target *wrt-1* and *wrt-10*. Our results suggest that the piRNA pathway and its downstream Hegdehog signaling encode the germline-emanated pro-aging signal.

### Patched receptors mediate mating-induced shrinking and aging

**Fig. 5:**
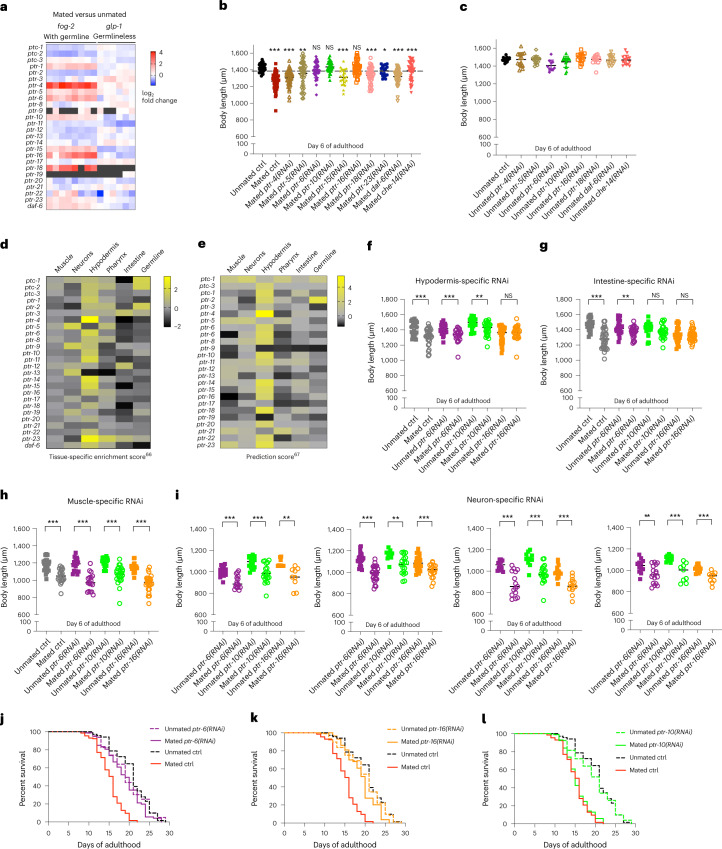
Somatic expression of *ptr-6* and *ptr-16* but not other Patched-related receptor-encoding genes is required for mating-induced death. **a**, Relative expression (mated versus unmated) of *ptr* homolog genes in worms with a functional germline (*fog-2*) and germlineless worms (*glp-1*). Larger fold changes indicate higher expression after mating. **b**, Body size measurements of mated worms with RNAi treatment of Patched receptor homolog genes. *fog-2(q71)* unmated (control RNAi), 1,426 ± 46 μm (*n* = 44); mated (control RNAi), 1,264 ± 99 μm (*n* = 48); *P* < 0.001; mated *ptr-4(RNAi)*, 1,305 ± 94 μm (*n* = 43); *P* < 0.001; mated *ptr-5(RNAi)*, 1,357 ± 146 μm (*n* = 45); *P* = 0.0032; mated *ptr-6(RNAi)*, 1,396 ± 91 μm (*n* = 42); *P* = 0.0502; mated *ptr-10(RNAi)*, 1,436 ± 65 μm (*n* = 34); *P* = 0.4623; mated *ptr-15(RNAi)*, 1,311 ± 96 μm (*n* = 22); *P* < 0.001; mated *ptr-16(RNAi)*, 1,422 ± 83 μm (*n* = 43); *P* = 0.7409; mated *ptr-18(RNAi)*, 1,337 ± 96 μm (*n* = 42); *P* < 0.001; mated *ptr-23(RNAi)*, 1,387 ± 65 μm (*n* = 17); *P* = 0.0104; mated *daf-6(RNAi)*, 1,320 ± 86 μm (*n* = 44); *P* < 0.001; mated *che-14(RNAi)*, 1,388 ± 112 μm (*n* = 44); *P* = 0.0394. *P < 0.05, **P < 0.01, ***P < 0.001. **c**, *ptr* homolog knockdown does not significantly affect the body size of unmated *fog-2* hermaphrodites (unmated control of **b**). Body size measurements: control, 1,474 ± 25 μm (*n* = 15); *ptr-4*, 1,473 ± 78 μm (*n* = 15); *ptr-5*, 1,472 ± 45 μm (*n* = 15); *ptr-6*, 1,406 ± 61 μm (*n* = 10); *ptr-10*, 1,448 ± 63 μm (*n* = 15); *ptr-16*, 1,491 ± 52 μm (*n* = 15); *ptr-18*, 1,474 ± 49 μm (*n* = 15); *daf-6*, 1,467 ± 48 μm (*n* = 15) and *che-14*, 1,468 ± 53 μm (*n* = 15). **d**,**e**, Tissue-specific expression prediction of Patched receptor homolog genes by the Tissue Expression Predictions for *C. elegans* program, version 1.0 (**d**) and the current version (**e**) (https://worm.princeton.edu/). **f**, Hypodermis-specific RNAi strain (DCL569) unmated control, 1,418 ± 94 μm (*n* = 27); mated control, 1,309 ± 104 μm (*n* = 32); *P* < 0.001; unmated *ptr-6(RNAi)*, 1,409 ± 62 μm (*n* = 29); mated *ptr-6(RNAi)*, 1,328 ± 84 μm (*n* = 23); *P* < 0.001; unmated *ptr-10(RNAi)*, 1,483 ± 78 μm (*n* = 21); mated *ptr-10(RNAi)*, 1,415 ± 85 μm (*n* = 30); *P* = 0.0055; unmated *ptr-16(RNAi)*, 1,340 ± 102 μm (*n* = 34); mated *ptr-16(RNAi)*, 1,359 ± 88 μm (*n* = 28); *P* = 0.4494. **g**, Intestine-specific RNAi strain (IG1839) unmated control, 1,472 ± 81 μm (*n* = 26); mated control, 1,282 ± 124 μm (*n* = 32); *P* < 0.001; unmated *ptr-6(RNAi)*, 1,433 ± 93 μm (*n* = 24); mated *ptr-6(RNAi)*, 1,372 ± 70 μm (*n* = 30); *P* = 0.0077; unmated *ptr-10(RNAi)*, 1,411 ± 85 μm (*n* = 26); mated *ptr-10(RNAi)*, 1,374 ± 85 μm (*n* = 27); *P* = 0.1190; unmated *ptr-16(RNAi)*, 1,328 ± 103 μm (*n* = 24); mated *ptr-16(RNAi)*, 1,327 ± 70 μm (*n* = 40); *P* = 0.9813. **h**, Muscle-specific RNAi strain (NR350) unmated control, 1,184 ± 77 μm (*n* = 23); mated control, 1,047 ± 75 μm (*n* = 23); *P*  < 0.001; unmated *ptr-6(RNAi)*, 1,183 ± 74 μm (*n* = 19); mated *ptr-6(RNAi)*, 979 ± 99 μm (*n* = 18); *P* < 0.001; unmated *ptr-10(RNAi)*, 1,210 ± 52 μm (*n* = 20); mated *ptr-10(RNAi)*, 1,064 ± 99 μm (*n* = 27); *P* < 0.001; unmated *ptr-16(RNAi)*, 1,148 ± 58 μm (*n* = 34); mated *ptr-16(RNAi)*, 966 ± 99 μm (*n* = 24); *P* < 0.001. **i**, *ptr* genes do not function in the neurons to regulate postmating body size. Four different neuron-specific RNAi strains were used: XE1474, XE1581, XE1582 and XE1375. Body size measurements: XE1474 unmated *ptr-6(RNAi)*, 1,004 ± 47 μm (*n* = 16); mated *ptr-6(RNAi)*, 903 ± 59 μm (*n* = 16); *P* < 0.001; unmated *ptr-10(RNAi)*, 1,087 ± 53 μm (*n* = 17); mated *ptr-10(RNAi)*, 985 ± 86 μm (*n* = 20); *P* < 0.001; unmated *ptr-16(RNAi)*, 1,080 ± 38 μm (*n* = 9); mated *ptr-16(RNAi)*, 946 ± 95 μm (*n* = 9); *P* = 0.0012. XE1581 unmated *ptr-6(RNAi)*, 1,119 ± 59 μm (*n* = 26); mated *ptr-6(RNAi)*, 987 ± 70 μm (*n* = 27); *P* < 0.001; unmated *ptr-10(RNAi)*, 1,159 ± 50 μm (*n* = 10); mated *ptr-10(RNAi)*, 1,068 ± 86 μm (*n* = 19); *P* = 0.0047; unmated *ptr-16(RNAi)*, 1,088 ± 67 μm (*n* = 23); mated *ptr-16(RNAi)*, 1,013 ± 62 μm (*n* = 19); *P* < 0.001. XE1582 unmated *ptr-6(RNAi)*, 1,055 ± 41 μm (*n* = 13); mated *ptr-6(RNAi)*, 882 ± 113 μm (*n* = 15); *P* < 0.001; unmated *ptr-10(RNAi)*, 1,111 ± 65 μm (*n* = 11); mated *ptr-10(RNAi)*, 975 ± 86 μm (*n* = 20); *P* < 0.001; unmated *ptr-16(RNAi)*, 995 ± 61 μm (*n* = 14); mated *ptr-16(RNAi)*, 865 ± 72 μm (*n* = 13); *P* < 0.001. XE1375 unmated *ptr-6(RNAi)*, 1,041 ± 63 μm (*n* = 12); mated *ptr-6(RNAi)*, 961 ± 82 μm (*n* = 17); *P* = 0.0096; unmated *ptr-10(RNAi)*, 1,114 ± 26 μm (*n* = 13); mated *ptr-10(RNAi)*, 980 ± 85 μm (*n* = 9); *P* < 0.001; unmated *ptr-16(RNAi)*, 1,015 ± 33 μm (*n* = 11); mated *ptr-16(RNAi)*, 932 ± 52 μm (*n* = 9); *P* < 0.001. **P < 0.01, ***P < 0.001. **j**–**l**, RNAi knockdown of *ptr-6* and *ptr-16* increases the lifespan of mated worms. Mated control, 15.1 ± 0.3 days, *n* = 257; unmated control, 20.6 ± 0.5 days, *n* = 99. **j**, Unmated *ptr-6(RNAi)*, 19.6 ± 0.6 days, *n* = 91; mated *ptr-6(RNAi)*, 18.8 ± 0.5 days, *n* = 114. Log-rank (Mantel–Cox) test revealed that the lifespan of mated *ptr-6(RNAi)* worms was significantly different from that of mated control worms (*P* < 0.001). Two-way ANOVA revealed that mating-induced lifespan decrease of *ptr-6(RNAi)* worms was significantly different from that of control worms (*P* = 0.0037). **k**, Unmated *ptr-16(RNAi)*, 19.8 ± 0.5 days, *n* = 105; mated *ptr-16(RNAi)*, 19.1 ± 0.5 days, *n* = 120. Log-rank (Mantel–Cox) test revealed that the lifespan of mated *ptr-16(RNAi)* worms was significantly different from that of mated control worms (*P* = 0.0214). Two-way ANOVA revealed that mating-induced lifespan decrease of *ptr-16(RNAi)* worms was significantly different from that of control worms (*P* = 0.0003). **l**, Unmated *ptr-10(RNAi)*, 19.7 ± 0.7 days, *n* = 100; mated *ptr-10(RNAi)*, 15.5 ± 0.3 days, *n* = 120. Log-rank (Mantel–Cox) test revealed that the lifespan of mated *ptr-10(RNAi)* worms was not significantly different from that of mated control worms (*P* = 0.0709). Two-way ANOVA revealed that mating-induced lifespan decrease of *ptr-10(RNAi)* worms was not significantly different from that of control worms (*P* = 0.5749).

**Fig. 6:**
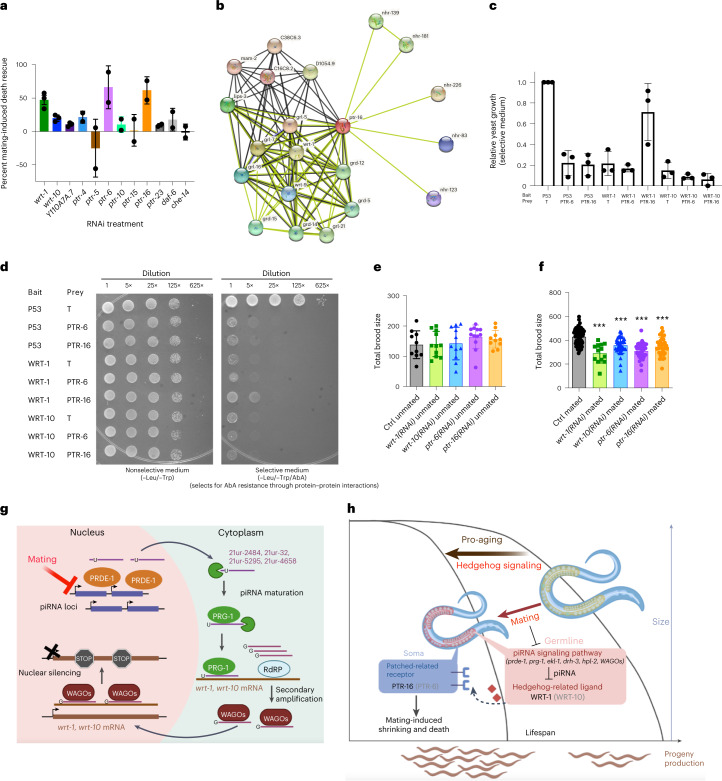
Hedgehog signaling encodes the germline-to-soma aging signal in mated hermaphrodites. **a**, Summary of the mating-induced death rescue effect of RNAi knockdown of individual Hedgehog signaling components. Each black dot represents one biological replicate of the lifespan assay. Data are presented as mean values of rescue effect ± s.d. (error bars). Three replicates of lifespan assays were performed for worms treated with *wrt-1*, *wrt-10* and *Y110A7A.7* RNAi. Two replicates of lifespan assays were performed for worms treated with *ptr-4*, *ptr-5*, *ptr-6*, *ptr-10*, *ptr-15*, *ptr-16*, *ptr-23*, *daf-6* and *che-14* RNAi. **b**, PTR-16 interaction network predicted by STRING. **c**, Relative yeast cell growth on selective medium (quantified from the fivefold dilution spots on yeast two-hybrid assay plates, see **d** and Methods for details). Data are presented as relative growth ± s.d. Three independent replicates of yeast two-hybrid assay were performed. **d**, Representative pictures of yeast two-hybrid assay. A dominant mutant version of the AUR1 gene that encodes the enzyme inositol phosphoryl ceramide synthase is expressed in the Y2HGold yeast strain in response to positive interaction between the bait and prey, conferring strong resistance to the otherwise highly toxic drug AbA. The final concentration of AbA on plates was 200 ng ml^–1^. Single colonies of each type were inoculated and allowed to grow overnight at 30 °C to reach stationary phase (OD > 1.5). From the culture, 20 μl of undiluted culture and 5×, 25×, 125× and 625× dilutions were spotted on the assay plates. Plates were imaged after incubating for 4 days at 30 °C. Quantification of yeast growth of the fivefold dilution spots is shown in **c**. Growth on selective medium was normalized to growth on nonselective medium first before comparing with the positive control spot (P53-T); Methods). **e**, Inhibiting Hedgehog signaling by RNAi does not affect the unmated (self-fertilized) brood size of the wildtype N2 hermaphrodites. N2 control unmated, 138 ± 46, *n* = 10; *wrt-1(RNAi)* unmated, 141 ± 42, *n* = 11; *wrt-10(RNAi)* unmated, 142 ± 54, *n* = 11; *ptr-6(RNAi)* unmated, 164 ± 39, *n* = 12; *ptr-16(RNAi)* unmated, 156 ± 29, *n* = 10. Data are presented as total brood size ± s.d. **f**, Inhibiting Hedgehog signaling by RNAi in mated N2 hermaphrodites leads to significantly reduced total mated brood size. Control mated, 429 ± 67, *n* = 65; *wrt-1(RNAi)* mated, 290 ± 81, *n* = 13; *wrt-10(RNAi)* mated, 358 ± 68, *n* = 41; *ptr-6(RNAi)* mated, 310 ± 65, *n* = 33; *ptr-16(RNAi)* mated, 345 ± 76, *n* = 38. Data are presented as total brood size ± s.d. ***P < 0.001: two-tailed unpaired *t*-test was used for total mated brood size comparison (all were compared with that of control mated worms). **g**, Model of the role of mating in piRNA-mediated regulation of *wrt-1* and *wrt-10* expression. **h**, Model of piRNA-mediated Hedgehog signaling as the germline-to-soma pro-aging signal in mated hermaphrodites.

To identify receptors for the Hedgehog ligands secreted from the germline upon mating, we tested the roles of ten Patched receptor homolog genes in shrinking after mating. These genes were chosen because their expression was induced by mating in a germline-dependent manner (Fig. 5a). Knocking down *ptr-6*, *ptr-10* and *ptr-16* prevented mated worms from shrinking (Fig. 5b,c), making them likely candidates to receive the germline-originating signal encoded by *wrt-1* and *wrt-10*. Most Hedgehog signaling receptors, including *ptr-6, ptr-10* and *ptr-16*, are predicted to be expressed in the hypodermis (Fig. 5d,e)^66,67^. Using tissue-specific RNAi strains, we found that *ptr-16* in the hypodermis, and *ptr-16* and *ptr-10* in the intestine, were required for mediating mating-induced shrinking (Fig. 5f,g); knockdown in muscle and neurons had no effect, ruling out these tissues as the Hedgehog signal-receiving tissues (Fig. 5h,i). Reducing *ptr-6* and *ptr-16*, but not *ptr-10* or the other Patched-related receptor genes, rescued mating-induced early death (Fig. 5j–l). *Ptr-6* and *ptr-16* RNAi yielded a similar or greater degree of protection compared with *wrt-1* RNAi in mated worms (Fig. 6a). Moreover, PTR-16 is predicted to interact with WRT-1 according to the STRING database (Fig. 6b), which integrates all publicly available sources of protein–protein interaction information^68^. To test this potential interaction, we performed yeast two-hybrid assays; a modest interaction between WRT-1 and PTR-16 was observed (Fig. 6c,d), suggesting that PTR-16 is most probably the receptor that receives the pro-aging Hedgehog WRT-1 signal from the germline upon mating.

Finally, we wondered whether there is any generational penalty for eliminating mating-induced shrinking and death, as these processes are deleterious to the mother. While *wrt-1*, *wrt-10*, *ptr-6* and *ptr-16* knockdown all produce unmated brood sizes similar to those of wildtype (Fig. 6e), knockdown of Hedgehog signaling components in mated worms led to a 20–30% reduction in brood size (Fig. 6f; *P* < 0.001). Therefore, while inhibiting germline piRNA-mediated Hedgehog signaling is beneficial to the mothers, as it prevents mated worms from shrinking and largely rescues mating-induced death, such benefits do not come without a cost to progeny. Signaling from the mated germline, while deleterious to the mother’s soma and ultimately leading to her death, may be an unavoidable cost of activation of the germline upon mating that is necessary for sufficient nutrient provisioning to progeny.

## Discussion

In animals across taxa, germline hyperactivity leads to accelerated aging^14^; however, the underlying mechanisms are poorly understood. Here we found that mating-induced piRNA downregulation in the germline releases suppression of the Hedgehog signaling pathway, which in turn leads to body size and lifespan decrease in mated worms (Fig. 6g,h). Our results suggest that piRNA-regulated Hedgehog signaling encodes the previously unknown and long-sought germline-to-soma pro-aging signal^7^.

The Hedgehog signaling pathway is a key regulator of animal development^69^; our study provides evidence for an additional critical role in adult postmating lifespan regulation in *C.* *elegans*, distinct from its role in developmental growth processes. Hedgehog signaling also participates in adult soma-to-germline communication: overexpression of hypodermal *wrt-10* delays reproductive decline and improves germline health^70^. Therefore, *wrt-10* could function as a feed-forward loop in mated worms, achieving sustained germline hyperactivity at the cost of exacerbated somatic collapse (that is, shrinking). Our results suggest that there is likely a trade-off between somatic integrity and progeny production in mated animals, as demonstrated by the reduced mated brood size of animals with reduced Hedgehog signaling (Fig. 6h).

The involvement of Hedgehog signaling in lifespan regulation is not limited to worms. Altered Hedgehog signaling reduces survival of *Drosophila* larvae under starvation^71^, impaired Hedgehog signaling in glia affects the lifespan of adult *Drosophila*^72^ and the Hedgehog pathway inhibitor saridegib dramatically increases lifespan by fourfold in a mouse medulloblastoma model^73^. Therefore, Hedgehog signaling seems to have an evolutionarily conserved role in lifespan regulation beyond its well-established function in development.

Our results unveil a new function for the piRNA pathway in transcriptional regulation, an underappreciated aspect of piRNA biology compared with its better-studied role in germline repression of transposable elements (TE)^74^. TE-independent piRNA functions in endogenous mRNA regulation of developmental processes include embryonic patterning, germ cell specification and stem cell biology^75^. Our results expand the roles of piRNA pathways in the regulation of gene expression to adult functions^59,76,77^. Here, we describe piRNA-mediated regulation of a known signaling pathway in the adult germline (Fig. 6g,h). These results also highlight the fact that piRNA expression is regulated and tunable by mating status.

Mating-induced germline-to-soma piRNA-mediated Hedgehog signaling elegantly coordinates germline function and somatic aging. In an unmated female with low or no germline proliferation, the priority of the germline is to maintain its integrity until mating occurs, while avoiding unnecessary proliferation or differentiation of the germ cells. piRNAs contribute to this process by suppressing errant TE expression and developmental programs, including those regulated by Hedgehog signaling. By contrast, upon mating, the germline switches to progeny production mode, and the previous suppression of Hedgehog signaling by piRNAs is released to allow rapid germline stem cell proliferation and further differentiation (Fig. 6g). As Hedgehog ligands are secreted, activated Hedgehog signaling may be co-opted as a germline-to-soma signal to coordinate the somatic responses to elevated germline activity. Postmating shrinking correlates with the reallocation of somatic resources to the germline^12,13,78^ and the reduction in energy devoted to somatic integrity maintenance, reflecting the direct cost of germline hyperactivity and substantially increased progeny production. Signaling from the mated germline, while deleterious to the mother’s soma and ultimately leading to her death, may be an unavoidable cost of activation of the germline upon mating that is necessary for sufficient nutrient provisioning to progeny. Our study reveals a mechanism that can efficiently convey germline status to the soma in adulthood, allowing the animals to better organize the balance between reproduction and somatic maintenance, optimizing reproductive fitness.

Of the three mating-related killing mechanisms—male pheromone, seminal fluid, and sperm activation of germline proliferation—the last has the highest chance of being evolutionarily conserved. Male pheromone-induced killing kills hermaphrodites only at nonphysiologically high concentrations, and kills males specifically but only in androdioecious species^13^. Seminal fluid kills the mother by turning off her IIS/FOXO protective pathway^12,13^, which is unnecessary for successful reproduction. By contrast, germline activation upon mating is the most likely to be both necessary and shared with other animals, as germline activity correlates with shorter lifespan in worms, flies, mice, and possibly humans^7–12,14^. Because germline proliferation may be induced by mating in other organisms, and piRNAs are ubiquitous in animal germlines, it will be interesting to see whether mating-induced piRNA regulation of signaling from germline to somatic tissues is conserved.

## Methods

### Strains

*C.* *elegans* strains used in the study were as follows:

**Table:** 

Strains	Genotype	Source
N2	Wildtype	CGC
CB4108	*fog-2(q71) V*	CGC
CB4037	*glp-1(e2141) III*	CGC
HC196	*sid-1(qt9) V*	CGC
CS1	*sma-9(wk55) X*	CGC
YY470	*dcr-1(mg375) III*	CGC
SX922	*prg-1(n4357) I*	CGC
SX2499	*prde-1(mj207) V*	CGC
VC3150	*ekl-1(ok1197) I/hT2 [bli-4(e937) let-?(q782) qIs48] (I;III)*	CGC
WM140	*drh-3(tm1217) I/hT2 [bli-4(e937) let-?(q782) qIs48] (I;III); him-8(e1489) IV*	CGC
RB995	*hpl-2(ok916) III*	CGC
WM191	*MAGO12 mutant. Full genotype: sago-2(tm894) ppw-1(tm914) ppw-2(tm1120) wago-2(tm2686) wago-1(tm1414) I; wago-11(tm1127) wago-5(tm1113) wago-4(tm1019) II; hrde-1(tm1200) sago-1(tm1195) III; wago-10(tm1186) V; nrde-3(tm1116) X*.	CGC
JH3225	*meg-3(tm4259) meg-4(ax2026) X*	CGC
JH3229	*meg-1(vr10) meg-3(tm4259) X*.	CGC
CQ655	*prg-1(n4357) I; unc-119(ed3) III; vrIs79[Ppie-1::GFP::prg-1* + *unc-119(+)]*	Murphy laboratory
CQ479	*rde-1(ne219); wqEx50[Pdpy-7::GFP; pY37A1B.5::GFP; Pdpy-7::rde-1::unc-54 3’UTR; PY37A1B.5::rde-1::unc-54 3’UTR]*	Murphy laboratory
DCL569	*mkcSi13 II; rde-1(mkc36) V*	CGC
IG1839	*frSi17 II; frIs7 IV; rde-1(ne300) V*	CGC
NR350	*rde-1(ne219) V; kzIs20*	CGC
XE1474	*wpSi6 II; eri-1(mg366) IV; rde-1(ne219) V; lin-15B(n744) X*.	CGC
XE1581	*wpSi10 II; eri-1(mg366) IV; rde-1(ne219) V; lin-15B(n744) X*	CGC
XE1582	*wpSi11 II; eri-1(mg366) IV; rde-1(ne219) V; lin-15B(n744) X*	CGC
AA278	*dhIs59 [Topo::daf-9::GFP* + *lin-15(+)]*	CGC
XE1375	*wpIs36 I; wpSi1 II; eri-1(mg366) IV; rde-1(ne219) V; lin-15B(n744) X*	CGC
WM300	*alg-4(ok1041) III; alg-3(tm1155) IV*	CGC
YY968	*znfx-1(gg544[3xflag::gfp::znfx-1]) II; pgl-1(gg547[pgl-1::3xflag::tagRFP]) IV*	CGC

### Germline dissection

Germline dissection was modified from a previous publication^79^. Worms were transferred to iced M9 buffer for dissection. Heads or tails were removed with 26G needles, allowing the distal portion of the germline to pop out of the worm. Glass capillaries pulled with an opening just large enough to fit the end of the germline were used to detach them rapidly at the ventral-to-distal bend. Dissected distal germlines were transferred immediately into 1.5 ml Eppendorf tubes filled with 500 μl Trizol. About 200 germlines were collected for each biological replicate.

### RNA isolation and library preparation

Tubes containing dissected germlines (immersed in Trizol) were put in an Eppendorf MixMate Vortex Mixer at 800 r.p.m. and 65 °C for 1 h before the isolation process. Total RNA was extracted from Trizol using the mirVana miRNA isolation kit (ThermoFisher). mRNA libraries for directional RNA-seq were prepared using the SMARTer Apollo System and were sequenced (150-nt single-end) on the Illumina NovaSeq platform. RNA samples for small RNA-seq were treated with 5′ polyphosphatase (Lucigen) and prepared using the SMARTer Apollo system with modifications for small RNA library preparation. Small RNA-containing libraries were Blue Pippin size selected (15–30 nt insert size) before 75-nt single-end sequencing.

### Transcriptome data analysis

#### RNA-seq

FASTQC was used to assess read quality scores. Universal adapter sequences were trimmed from small RNA library sequences using Cutadapt. Reads were mapped to the *C.* *elegans* genome (UCSC February 2013, ce11/ws245) using Bowtie. Count matrices were generated using featureCounts. Data were normalized with a variance-stabilizing transformation (DESeq2). DESeq2 was used for differential expression analysis. Genes at *P*_adj _< 0.05 were considered significantly differentially expressed. PCA was carried out using the R method (prcomp). Heatmaps were generated in R using normalized read counts (variance-stabilizing transformation). Tissue enrichment and gene ontology (GO) term enrichment analysis were performed using wormbase enrichment analysis (https://wormbase.org/tools/enrichment/tea/tea.cgi) for significantly differentially-expressed genes. Predicted targets of piRNAs were retrieved from piRTarBase (http://cosbi6.ee.ncku.edu.tw/piRTarBase/) using the default ‘relaxed’ piRNA targeting setting, see Supplementary Data 2 for the full list. Sequences were deposited at NCBI BioProject PRJNA892981.

#### Microarrays

Microarray data of mated and unmated *glp-1(e2141)* was retrieved from a previous publication^34^. Microarray data of mated and unmated *fog-2(q71)* was retrieved from a previous publication^61^. Hermaphrodites were mated on Day 1 of adulthood for 24 h in a 2:1 male:hermaphrodite ratio. About 200 hermaphrodites were collected on Day 2/3 of adulthood for each biological replicate. RNA was extracted by the heat vortexing method. Two-color Agilent microarrays were used for expression analysis. Significantly differentially-expressed gene sets were identified using significance analysis of microarrays (SAM)^80^. One-class SAM was performed to identify genes that are significantly differentially expressed after mating. The lists were then compared with significantly upregulated genes in dissected germline (RNA-seq).

### Lifespan

NGM plates (60 mm) plates were used to set up group mating. Each 60 mm NGM plate was seeded with OP50 to make a bacterial lawn of around 3 cm in diameter 2 days before mating. All lifespan assays were performed at room temperature (20–21 °C). About 50 hermaphrodites and 100 young (Day 1−2 of adulthood) males were transferred onto the plate. Then, 24–48 h later, the hermaphrodites were transferred onto newly seeded 60 mm NGM plates in the absence of males for lifespan assays. No 5-fluro-2′-deoxyuridinevwas added to the plates. We confirmed successful mating for all worms by checking their progeny. With successful mating, about half of the progeny are male. For the only two sterile strains used in this study, *ekl-1* and *drh-3* homozygous mutants, we confirmed successful mating empirically by observing the decrease in darkness of mated sterile worms under normal light, which indicates mating-induced fat loss. About 25 synchronized Day 1 hermaphrodites were transferred onto each plate. The hermaphrodites were transferred daily onto new seeded plates in the first week of the lifespan assay. Afterward, they were transferred once every 2 days. When RNAi was used in lifespan assay, RNAi treatment always started from eggs for all the experiments in this study. Kaplan–Meier analysis with log-rank (Mantel–Cox) method was performed to compare the lifespans of different groups. ‘Bagged’ worms were censored on the day of the event.

### Body size measurements

Mating set up was the same as described in Lifespan. We confirmed successful mating for all the worms by checking their progeny. With successful mating, about half of the progeny are male. For the only two sterile strains used in this study, *ekl-1* and *drh-3* homozygous mutants, we confirmed the successful mating empirically by observing the decrease in darkness of mated sterile worms under normal light, which indicates mating-induced fat loss. Images of live hermaphrodites on 60 mm plates were taken on Day 6/7 of adulthood with a Nikon SMZ1500 microscope. When RNAi was used, RNAi treatment always started from eggs for all the experiments. ImageJ was used to analyze the body size of the worms. The middle line of each worm was delineated using the segmented line tool and the total length was documented as the body length of the worm. A *t*-test was performed to compare the body size differences between groups of worms in the same day.

### Brood size

Individual hermaphrodites after mating were transferred onto 3 cm NGM plates seeded with 25 ml OP50 and moved to fresh plates daily until reproduction ceased. The old plates were left at 20 °C for 2 days to allow the offspring to grow into adults, which were counted manually for daily production and total brood size. Between 10 and 25 plates of individual worms of each genotype per treatment were counted to account for individual variation.

### P granule imaging and quantification

mCherry-tagged fluorescent PGL-1 was visualized in living nematodes (YY968) by mounting young adult animals on 2% agarose pads with M9 buffer with 20 mM levamisole. Fluorescent images were captured using a Nikon Ti microscope with a ×100 objective. Images were processed and quantified in ImageJ. The quantification of germline granule fluorescence was performed using ImageJ. For every image, a region of interest (ROI) with a clear focus of P granules was selected manually. The area of the whole ROI was kept the same for all images. The number of puncta within the ROI was measured blindly for each germline and image. The densities of germline P granules were calculated as: the number of puncta within the ROI per the area of the whole ROI. Densities of germline granules were determined for 50–60 ROIs, and the mean and s.d. were calculated using GraphPad Prism. A *t*-test was performed to compare the P granule density differences between mated and unmated germlines.

### Germline piRNA overexpression

We modified recently developed piRNAi^65^ to overexpress endogenous piRNAs. The scaffold information was obtained from the website https://www.wormbuilder.org/piRNAi/. Cluster was generated using simple search option with the default setting (targeted gene: *wrt-1* or *wrt-10*). Synthetic piRNAs were replaced by endogenous piRNAs as indicated by the text and figure legends. The sequences (about 1.5 kb) were synthesized by Twist Bioscience. The injection mix consisted of 20 ng μl^–1^ synthetic dsDNA piRNA overexpression cluster with adapters that were not further purified (Twist Bioscience), 2 ng μl^–1^ coinjection marker (Pmyo2::mCherry::unc-54 3′ UTR), and 1 kb DNA ladder to a final total DNA concentration of 100 ng μl^–1^. F1 progeny with red pharynx were selected from injected animals. Synchronized F2 progeny from positive transgenic F1 worms were used in mating and body size measurement assays.

The sequences of piRNA overexpression clusters used in this study are as follows:

piRNA overexpression cluster targeting *wrt-1* (uppercase: piRNAs)

cgcgcttgacgcgctagtcaactaacataaaaaaggtgaaacattgcgaggatacatagaaaaaacaatacttcgaattcatttttcaattacaaatcctgaaatgtttcactgtgttcctataagaaaacattgaaacaaaatattaagtTAGAACTTCATCTTTAGAACActaattttgattttgattttgaaatcgaatttgcaaatccaattaaaaatcattttctgataattagacagttccttatcgttaattttattatatctatcgagttagaaattgcaacgaagataatgtcttccaaatactgaaaatttgaaaatatgtttttatggcaggtgctgacggattgccagaactcaaaatatgaaatttttatagttttgttgaaacagtaagaaaatcttgtaattactgtaaactgtttgctttttttaaagtcaacctacttcaaatctacttcaaaaattataatgtttcaaattacataactgtgtgaagttgggcgcccagttgtactgtagagcttcaatgttgataagatttattaacacagtgaaacaggtaatagttgtttgttgcaaaatcggaaatctctacatttcatatggtttttaattacaggtttgttttataaaataattgtgtgatggatattattttcagacctcatactaatctgcaaaccttcaaacaatatgtgaagtctactctgtttcactcaaccattcatttcaatttggaaaaaaatcaaagaaatgttgaaaaattttcctgtttcaacattatgacaaaaatgttatgattttaataaaaacaatTGTCATAAACGTAGAATCATCttctgtttttcttagaagtgttttccggaaacgcgtaattggttttatcacaaatcgaaaacaaacaaaaatttttttaattatttctttgctagttttgtagttgaaaattcactataatcatgaataagtgagctgcccaagtaaacaaagaaaatttggcagcggccgacaactaccgggttgcccgatttatcagtggaggatctacaaggctaactgcgttatctaatgtgatgtacacggttttcatttaaaaacaaattgaaacagaaatgactacattttcaaattgtctatttttgctgtgtttattttgccaccaacaatgagataatgtgttagccttgtcaatctagtaaactcacttaatgcaattcctccagccacatatgtaaacgttgtatacatgcagaaaacggttttttggttttaatgggaacttttgacaaattgttcgaaaatcttaagctgtcccatttcagttgggtgatcgattt

piRNA overexpression cluster targeting *wrt-10* (uppercase: piRNAs)

cgcgcttgacgcgctagtcaactaacataaaaaaggtgaaacattgcgaggatacatagaaaaaacaatacttcgaattcatttttcaattacaaatcctgaaatgtttcactgtgttcctataagaaaacattgaaacaaaatattaagtTAAATGAAAAGCTGGCTATGGctaattttgattttgattttgaaatcgaatttgcaaatccaattaaaaatcattttctgataattagacagttccttatcgttaattttattatatctatcgagttagaaattgcaacgaagataatgtcttccaaatactgaaaatttgaaaatatgtttttatggcaggtgctgacggattgccagaactcaaaatatgaaatttttatagttttgttgaaacagtaagaaaatcttgtaattactgtaaactgtttgctttttttaaagtcaacctacttcaaatctacttcaaaaattataatgtttcaaattacataactgtgtgaagttgggcgcccagttgtactgtagagcttcaatgttgataagatttattaacacagtgaaacaggtaatagttgtttgttgcaaaatcggaaatctctacatttcatatggtttttaattacaggtttgttttataaaataattgtgtgatggatattattttcagacctcatactaatctgcaaaccttcaaacaatatgtgaagtctactctgtttcactcaaccattcatttcaatttggaaaaaaatcaaagaaatgttgaaaaattttcctgtttcaacattatgacaaaaatgttatgattttaataaaaacaatTGGAATAGCGTAAACAAAAGAttctgtttttcttagaagtgttttccggaaacgcgtaattggttttatcacaaatcgaaaacaaacaaaaatttttttaattatttctttgctagttttgtagttgaaaattcactataatcatgaataagtgagctgcccaagtaaacaaagaaaatttggcagcggccgacaactaccgggttgcccgatttatcagtggaggatctacaaggctaactgcgttatctaatgtgatgtacacggttttcatttaaaaacaaattgaaacagaaatgactacattttcaaattgtctatttttgctgtgtttattttgccaccaacaatgagataatgtgttagccttgtcaatctagtaaactcacttaatgcaattcctccagccacatatgtaaacgttgtatacatgcagaaaacggttttttggttttaatgggaacttttgacaaattgttcgaaaatcttaagctgtcccatttcagttgggtgatcgattt

piRNA overexpression cluster control (uppercase: piRNAs)

cgcgcttgacgcgctagtcaactaacataaaaaaggtgaaacattgcgaggatacatagaaaaaacaatacttcgaattcatttttcaattacaaatcctgaaatgtttcactgtgttcctataagaaaacattgaaacaaaatattaagtTAGAAACTGATCTCTGAAAGTctaattttgattttgattttgaaatcgaatttgcaaatccaattaaaaatcattttctgataattagacagttccttatcgttaattttattatatctatcgagttagaaattgcaacgaagataatgtcttccaaatactgaaaatttgaaaatatgttACTTTCCATAACGTCGACAAAattgccagaactcaaaatatgaaatttttatagttttgttgaaacagtaagaaaatcttgtaattactgtaaactgtttgctttttttaaagtcaacctacttcaaatctacttcaaaaattataatgtttcaaattacataactgtgtAATTCGGGAGTCCTAATTCTAactgtagagcttcaatgttgataagatttattaacacagtgaaacaggtaatagttgtttgttgcaaaatcggaaatctctacatttcatatggtttttaattacaggtttgttttataaaataattgtgtgatggatattattttcagacctcatactaatctgcaaaccttcaaacaatatgtgaagtctactctgtttcactcaaccattcatttcaatttggaaaaaaatcaaagaaatgttgaaaaattttcctgtttcaacattatgacaaaaatgttatgattttaataaaaacaatGAAAATTTTGCTGAACACCTTttctgtttttcttagaagtgttttccggaaacgcgtaattggttttatcacaaatcgaaaacaaacaaaaatttttttaattatttctttgctagttttgtagttgaaaattcactataatcatgaataagtgagctgcccaagtaaacaaagaaaatttggcagcggccgacaactaccgggttgcccgatttatcagtggaggaGATCGAGGCTTAATGAACGGAatctaatgtgatgtacacggttttcatttaaaaacaaattgaaacagaaatgactacattttcaaattgtctatttttgctgtgtttattttgccaccaacaatTAAAAGTGGCTCCGAGCTAGGtcaatctagtaaactcacttaatgcaattcctccagccacatatgtaaacgttgtatacatgcagaaaacggttttttggttttaatgggaacttttgacaaattgttcgaaaatcttaagctgtcccatttcagttgggtgatcgattt

### Adapter sequences

Twist Primer Set 2 Fw: 5′-CAATCCGCCCTCACTACAACCG-3′

Twist Primer Set 2 Rev: 5′-TCCCTCATCGACGCCAGAGTAG-3′

### Yeast two-hybrid assay

Yeast two-hybrid assay was performed using Takara Matchmaker Gold Yeast Two-Hybrid System (catalog no. 630489). WRT-1 and WRT-10 cDNA were cloned in frame into the bait construct pGBKT7, whereas PTR-6 and PTR-16 cDNA were cloned in frame into the prey construct pGADT7. pGBKT7-53, which encodes the Gal4 DNA-BD fused with murine p53, and pGADT7-7, which encodes the Gal4 AD fused with SV40 large T-antigen, were used as positive interaction controls in the assay. The bait constructs and the prey constructs were cotransformed into Y2HGold yeast strain in various combinations to test potential interaction. A dominant mutant version of the AUR1 gene that encodes the enzyme inositol phosphoryl ceramide synthase is expressed in Y2HGold Yeast strain in response to positive interaction between the bait and prey, conferring strong resistance to the otherwise highly toxic drug Aureobasidin A (AbA). The final concentration of AbA used in the assay was 200 ng ml^–1^. Single colonies of each type were inoculated and allowed to grow overnight at 30 °C to reach stationary phase (OD > 1.5). From the culture, 20 μl of undiluted, 5×, 25×, 125× and 625× dilutions were spotted on the assay plates. Plates were imaged after incubating for 4 days at 30 °C. Quantification of yeast growth was performed according to the published protocol^81^.

### Tissue expression prediction

Tissue enrichment prediction analyses for ptr genes were performed using two methods: (1) the Tissue Expression Predictions for *C. elegans* program, v.1.0 (http://worm-tissue.princeton.edu/search/multi). The tissue-specific enrichment scores were used to generate the heatmap in Fig. 5d. Genes bulk query for their predicted tissue expression (https://worm.princeton.edu/). The prediction scores of principal tissues were used to generate the heatmap in Fig. 5e.

### Intestinal barrier function assay (Smurf assay)

The intestinal barrier function assay (Smurf assay) was performed according to the published protocol^82^ with a few modifications. Briefly, synchronized Day 1 worms were set up for mating. About 50 worms were removed from the mating plate or the control plate 48 h later, and suspended overnight in liquid cultures of OP50 bacteria mixed with blue dye (bromophenol blue, 0.4 g 100 ml^–1^ M9). Animals were then washed three times with M9 buffer before imaging using Nikon Ti with RGB illumination under ×10 or ×20 magnification.

### TE expression quantification

In addition to grouped germline samples collected for mRNA-seq and small RNA-seq, we collected single distal germline (four for each condition) into 10 μl 0.2% Triton X100 with RNase inhibitor (final concentration 1 U μl^–1^). mRNA libraries for directional RNA-seq were prepared using the SMARTer Apollo System and were sequenced (150-nt single-end) on the Illumina NovaSeq platform. Reads from germline mRNA-seq were mapped to the *C. elegans* RepBase database (https://www.girinst.org/server/RepBase/protected/repeatmaskerlibraries/RepBaseRepeatMaskerEdition-20181026.tar.gz) using Bowtie. Raw counts were then normalized on the total number of mapping reads and multiplied by 1,000,000, obtaining expression values indicated as reads per million mapped reads (RPM). Transposon type was determined according to RepBase’s annotation.

### Statistics and reproducibility

No statistical method was used to predetermine sample size. Sample sizes were chosen according to professional standards of the field for individual assays. For RNA-seq, three biological replicates were performed for each condition per genotype^83^. About 200 distal germlines were collected in each replicate^84^. No RNA-seq library was excluded from the analyses. In lifespan assays, each group contains 50–100 worms and at least two biological replicates were performed for each condition and genotype^85^. To compare body size changes, 30–50 worms per genotype per condition were measured^12^.

Reported results were consistently replicated across multiple experiments. Postmating body size measurements and lifespan assays were repeated twice if mating causes significant shrinking and early death. In the case of treatments that brought protection against mating-induced shrinking or early death, at least three replicates were performed to confirm the effect. During initial RNAi screen of candidates, the names of the targeting gene were blinded. The investigators were not blinded in follow-up lifespan assays, since only two to three groups of worms were tested each time. However, for these follow-up lifespan assays, the number of worms for each group was increased from around 50 (in blinded lifespan assays) to over 100. When quantifying the P granule intensities of mated and unmated worms, images were scored blindly. No randomization was necessary for this study because mated and unmated worms of specific genotype/treatment were always compared with each other.

Lifespan data were plotted as Kaplan–Meier survival curves and statistical analyses were performed using the log-rank (Mantel–Cox) test and two-way ANOVA. For body size measurements and fluorescence intensity quantification, two-tailed *t*-test was used for all comparisons to determine statistical significance in this study. Detailed information of each assay can be found in the corresponding figure legends.

Source Data for sequencing data and statistics displayed in the main figures are provided as Supplementary Files.

### Reporting summary

Further information on research design is available in the Nature Portfolio Reporting Summary linked to this article.

## Supplementary information

Reporting Summary

Supplementary Table 1. List of germline-specific upregulated genes that are also upregulated in mated *fog-2* whole worms with intact germlines.

Supplementary Table 2. List of predicted targets of 148 downregulated piRNAs in the mated germline.

Supplementary Table 3. List of piRNA-targeted germline-specific mating-induced genes (genes in Supplementary Data 1 that belong to the predicted targets of piRNAs in Supplementary Data 2).

## Data Availability

All RNA-seq reads are available on NCBI Sequence Read Archive (PRJNA892981). The microarray data were retrieved from previous publications^34,61^ and are available at http://puma.princeton.edu. All other data supporting the findings of this study are available from the corresponding author upon request. Source data are provided with this paper.

## Source data

Source Data Fig. 1. List of significantly differentially-expressed genes identified from mated versus unmated *fog-2* germline mRNA_seq (DESeq2 with Bonferroni adjustment of *P* values).
Source Data Fig. 2. **a**–**c**, List of significantly differentially-expressed genes identified from mated versus unmated *fog-2* germline small RNA-seq (DESeq2 with Bonferroni adjustment of *P* values). **f**, Summary of TE mapped reads from germline mRNA-seq.
Source Data Fig. 3. List of significantly differentially-expressed genes identified from mated versus unmated *prde-1* germline mRNA-seq (DESeq2 with Bonferroni adjustment of *P* values).
Source Data Fig. 4. List of germline-specific upregulated genes (subtracting the genes that are upregulated in mated *glp-1* germlineless worms^34^ identified by SAM from those significantly upregulated genes in mated germlines in Source Data for Fig. 1g,h.
