# Supplementary material for: piRNAs regulate a Hedgehog germline-to-soma pro-aging signal
Source: Nat Aging. 2023 Jan 9;3(1):47–63. doi: 10.1038/s43587-022-00329-2 (PMC10154208; doi:10.1038/s43587-022-00329-2)
Supplement: Supplementary file 1 — Reporting Summary [file 43587_2022_329_MOESM1_ESM.pdf]

nature portfolio

Corresponding author(s): Coleen T. Murphy

Last updated by author(s): Oct 21, 2022

Reporting Summary

Nature Portfolio wishes to improve the reproducibility of the work that we publish. This form provides structure for consistency and transparency in reporting. For further information on Nature Portfolio policies, see our Editorial Policies and the Editorial Policy Checklist.

Statistics

For all statistical analyses, confirm that the following items are present in the figure legend, table legend, main text, or Methods section.

n/a	Confirmed	
☐	☒	The exact sample size (*n*) for each experimental group/condition, given as a discrete number and unit of measurement
☐	☒	A statement on whether measurements were taken from distinct samples or whether the same sample was measured repeatedly
☐	☒	The statistical test(s) used AND whether they are one- or two-sided<br>*Only common tests should be described solely by name; describe more complex techniques in the Methods section.*
☒	☐	A description of all covariates tested
☒	☐	A description of any assumptions or corrections, such as tests of normality and adjustment for multiple comparisons
☐	☒	A full description of the statistical parameters including central tendency (e.g. means) or other basic estimates (e.g. regression coefficient) AND variation (e.g. standard deviation) or associated estimates of uncertainty (e.g. confidence intervals)
☐	☒	For null hypothesis testing, the test statistic (e.g. *F*, *t*, *r*) with confidence intervals, effect sizes, degrees of freedom and *P* value noted<br>*Give P values as exact values whenever suitable.*
☒	☐	For Bayesian analysis, information on the choice of priors and Markov chain Monte Carlo settings
☒	☐	For hierarchical and complex designs, identification of the appropriate level for tests and full reporting of outcomes
☒	☐	Estimates of effect sizes (e.g. Cohen's *d*, Pearson's *r*), indicating how they were calculated

Our web collection on statistics for biologists contains articles on many of the points above.

Software and code

Policy information about availability of computer code

Data collection	No software was used for data collection.
Data analysis	n/a

For manuscripts utilizing custom algorithms or software that are central to the research but not yet described in published literature, software must be made available to editors and reviewers. We strongly encourage code deposition in a community repository (e.g. GitHub). See the Nature Portfolio guidelines for submitting code & software for further information.

Data

Policy information about availability of data

All manuscripts must include a data availability statement. This statement should provide the following information, where applicable:

- Accession codes, unique identifiers, or web links for publicly available datasets
- A description of any restrictions on data availability
- For clinical datasets or third party data, please ensure that the statement adheres to our policy

All RNA sequencing datasets are deposited at NCBI BioProject BioProject ID PRJNA892981. We have made the request to make the datasets publicly available.

Field-specific reporting

Please select the one below that is the best fit for your research. If you are not sure, read the appropriate sections before making your selection.

☒ Life sciences ☐ Behavioural & social sciences ☐ Ecological, evolutionary & environmental sciences

For a reference copy of the document with all sections, see nature.com/documents/nr-reporting-summary-flat.pdf

Life sciences study design

All studies must disclose on these points even when the disclosure is negative.

Sample size	Sample sizes were chosen according to professional standards of the field for individual assays. For RNA-sequencing, three biological replicates were performed for each condition/genotype (Amrit and Ghazi 2017). About 200 distal germlines were collected in each replicate (Robert et al., 2020). In lifespan assays, each group contains 50-100 worms, at least two biological replicates were performed for each condition/genotype (Shaw et al., 2007). To compare body size changes, 30-50 worms per genotype/condition were measured (Shi and Murphy, 2014). Detailed information regarding sample sizes of lifespan assays and body size measurements can be found in figure legends.
Data exclusions	Data were not excluded from analysis.
Replication	Reported results were consistently replicated across multiple experiments. Most experiments (body size measurements and lifespan assays) were repeated twice if mating causes significant shrinking and early death. In the case of treatments which brought protection against mating-induced shrinking or early death, at least three replicates were performed to confirm the effect.
Randomization	No randomization was necessary for this study because mated and unmated worms of specific genotype/treatment were always compared with each other.
Blinding	During initial RNAi screen of candidates, the names of the targeting gene were blinded. The investigators were not blinded in follow-up lifespan assays, since only two-three groups of worms were tested each time. However, for these follow-up lifespan assays, the number of worms for each group was increased from around 50 (in blinded lifespan assays) to over 100. When quantifiying the P granule intensities of mated and unmated worms, images were scored blindly.

Reporting for specific materials, systems and methods

We require information from authors about some types of materials, experimental systems and methods used in many studies. Here, indicate whether each material, system or method listed is relevant to your study. If you are not sure if a list item applies to your research, read the appropriate section before selecting a response.

Materials & experimental systems

n/a	Involved in the study	
☒	☐	Antibodies
☒	☐	Eukaryotic cell lines
☒	☐	Palaeontology and archaeology
☐	☒	Animals and other organisms
☒	☐	Human research participants
☒	☐	Clinical data
☒	☐	Dual use research of concern

Methods

n/a	Involved in the study	
☒	☐	ChIP-seq
☒	☐	Flow cytometry
☒	☐	MRI-based neuroimaging

Animals and other organisms

Policy information about studies involving animals; ARRIVE guidelines recommended for reporting animal research

Laboratory animals	C. elegans lab strains were used in this study. Detailed information (strain name, genotype, and sources) regarding each strain can be found in Material and Methods section. All the body size and lifespan experiments were performed using hermaphrodites. Young males (Day 1 - Day 2 of adulthood) were used only for mating. Hermaphrodites were mated on Day 1 of adulthood for 2 days and were separated from males on Day 3 and monitored throughout their lifespan.
Wild animals	This study did not involve wild animals.
Field-collected samples	No field collected samples were used in the study.
Ethics oversight	This study did not require an ethical approval.

Note that full information on the approval of the study protocol must also be provided in the manuscript.
